# Specific gene expression patterns associated as reliable biomarkers for predicting dental implant successful osseointegration: a literature review and focused meta-analysis

**DOI:** 10.3389/fphys.2025.1682440

**Published:** 2026-01-05

**Authors:** Jesus Alejandro Serrato-Pedrosa, Virgilio Bocanegra-García, Ignacio Villanueva-Fierro, Absalom Zamorano-Carrillo, Erwing Irving Rendón-Ramírez, Verónica Loera-Castañeda

**Affiliations:** 1 CIIDIR-Durango, Instituto Politécnico Nacional, Durango, Mexico; 2 Centro de Biotecnología Genómica, Instituto Politécnico Nacional, Reynosa, Tamaulipas, Mexico; 3 Laboratorio de Biofísica Computacional, Doctorado en Biotecnología, SEPI-ENMH Instituto Politécnico Nacional, Mexico City, Mexico; 4 Vivental, Grupo Odontológico., Durango, Mexico

**Keywords:** gene expression, osseointegration, implantogenomics, surface modifications, biomarkers

## Abstract

**Introduction:**

Scientific understanding of dental implant success has evolved significantly. Nowadays, it is well established that the long-term stability of an implant relies on osseointegration, a complex biological process directed by molecular and genetic signals at the bone-implant interface. This systematic review research synthesizes the recent scientific literature to identify specific genes and expression patterns that can indicate implant outcomes. Hence, the systematic review examines key signaling pathways, the influence of implant surface characteristics on cellular responses, and the potential for patient-specific therapeutic strategies.

**Methods:**

For this synthesis, relevant studies published between January 2020 and May 2025 were identified using the MEDLINE (via PubMed), Scopus and Web of Science databases, along the PRISMA methodology was employed. Furthermore, a quantitative meta-analysis was performed on a subset of homogenous in vitro studies.

**Results:**

The collected evidence reveals a distinct molecular signature for successful integration, initiated by the increased expression of primary bone-regulating genes, such as *RUNX2* and followed by the production of essential bone matrix proteins. In contrast, implant failure and peri-implantitis show a consistent association with a malfunctioning inflammatory response. This state is marked by elevated concentrations of inflammatory messengers (*IL-1β*, *IL-6*, and *TNF-α*) and an imbalanced *RANKL/OPG* ratio that favors bone resorption. Crucially, the implant surface is not a passive component in this process, its micro and nanoscale features are shown to actively guide these genetic pathways and shape the resulting cellular behavior. The findings revealed that modified implant surfaces significantly upregulate the expression of the key osteogenic transcription factor *RUNX2* (Standardized Mean Difference: 2.58; 95% CI: 1.21 to 3.95; p < 0.001).

**Discussion:**

The central conclusion is that specific, measurable gene expression patterns show promise as potential indicators of the biological processes governing dental implant outcomes. The emerging paradigm of implantogenomics aims to enable clinicians to perform personalized risk assessments and utilize advanced implant technologies to design individual, unique biological profile therapies and strategies, thereby optimizing the potential for long-term clinical success.

## 1 Introduction

The success of dental implants relies on long-term osseointegration, the process by which the implant integrates with the surrounding bone tissue (Alghamdi and Jansen, 2020). In the evaluation of clinical outcomes in implant dentistry, it is critical to distinguish between implant survival and implant success. The concept of implant survival refers to the outcome of the implant remaining (physically present in the bone tissue), regardless of its clinical condition. In contrast, implant success is a multifactorial measure, defined by a set of clinical criteria including implant stability (absence of mobility), no evidence of peri-implant radiolucency, minimal marginal bone loss over time, and the absence of signs and symptoms such as pain, infection, or paresthesia. This review focuses on the molecular biomarkers associated with the biological processes of osseointegration that meet both initial implant survival and the achievement of long-term clinical success. The constant advancement of omics technologies has provided a comprehensive approach to investigating health and disease molecular mechanisms. This facilitates the discovery of novel biomolecules and the interpretation of complex biological pathways (Ivanovski et al., 2022). The employment of these techniques paves the way to precision medicine, leading to key predictions in oral rehabilitation because it can adapt the procedure to align with the patient’s biological requirements (Bornes R. S. et al., 2023; Rakic et al., 2021). Recent research has focused on identifying reliable biomarkers to predict implant success and guide clinical decision-making. Various studies have indicated that specific genes and their expression patterns, including microRNAs (miRNAs) and differentially expressed messenger RNAs (mRNAs), as they play crucial roles in bone metabolism and can be predictive indicators of osseointegration success (Freitag et al., 2023). In this regard, systemic conditions such as cardiovascular diseases, obesity, and diabetes may also affect the expression of miRNAs, which could influence the outcomes of dental implant procedures. The activity of miRNA-142-3p and miRNA-146a has supported this, and their expression patterns reflect the health status of periodontal tissues (Asa’ad et al., 2020). Gene expression related to osteoblast differentiation and bone formation is critical for successful osseointegration. This has been established as a parameter in the inhibition of Osteopontin (*OPN*) and Osteonectin (*OCN*) genes, along with the quality of the surrounding bone and the host’s systemic health (Pandey et al., 2022; Makishi et al., 2022). Recent studies have highlighted the expression levels of Runt-related transcription factor 2 (*RUNX2*), Osteocalcin, and osteopontin in Human Mesenchymal Stem Cells (hMSCs) on various implant surfaces. The application of novel bioactive surfaces employing reduced Graphene Oxide (rGO) coated implants (R-ST group) exhibited significantly higher expression levels of these osteogenic markers than control groups, indicating their potential as reliable biomarkers for predicting successful osseointegration (Shin et al., 2022). On the contrary, some other studies have focused on analyzing the genetic factors associated with early implant failures and peri-implantitis, such as the *IL-1* genotype, which can significantly affect the acceptance of osseointegrated implants. Moreover, understanding the modulation of immunoinflammatory responses through gene expression is critical for the success of dental implants. The need for a balanced inflammatory response is critical in the regulation process, which involves specific genes that can either promote healing or contribute to implant failure (Gulati et al., 2023). The chemokine (*CCL5*) overexpression is present in incomplete bone-to-implant contact (BIC) areas. This finding suggests that *CCL5* may play a role in the inflammatory response surrounding implants and could work as a potential biomarker for evaluating the risk of poor osseointegration (Lechner et al., 2024). The identification of cytokines serves as a potential biomarker to anticipate complications. The strong expression of pro-inflammatory cytokines (*IL-1β*, *TNF-α*, *IL-6*, *IL-8*, and *IL-10*) is associated with the early stages of osseointegration. Elevated levels of *IL-6* and *IL-8* in the first 2 weeks post-implantation were positively correlated with early implant failure, suggesting their potential as biomarkers for predicting osseointegration success (Baker et al., 2024). Hence, these markers can indicate ongoing biological processes that may lead to implant failure. Thus, function as important diagnostic tools. Identifying such biochemical markers can aid in the development of targeted therapies and the effective monitoring of treatment responses (Bornes R. et al., 2023).

Likewise, the study of abnormal genetic processes in dental implant acceptance has extended to the significant role of microRNAs (miRNAs), which aid in regulating bone metabolism and osseointegration through post-transcriptional regulation, influencing gene expression related to osteogenic differentiation and bone remodeling. This is achieved through bioinformatic analysis of RNA sequencing data. These genes are implicated in various pathways, including extracellular matrix receptor interactions and the Phosphatidylinositol-3-Kinase (*PI3K*)-Protein Kinase B (*Akt*) signaling pathway (Wang et al., 2021a). Evaluating molecular expression extends beyond identifying the crucial genes involved in forming new bone and determining the essential ones for bone maintenance, repair, and remodeling. The Osteoclastogenesis activity is mainly regulated by the *TRAP* (Tartrate-Resistant Acid Phosphatase) indicating its potential role in bone resorption processes associated with implant failure, *TNF-alpha* (Tumor Necrosis Factor Alpha) inflammatory response, *RANK* (Receptor Activator of Nuclear Factor Kappa-B) differentiating osteoclast activity and *OPG* (Osteoprotegerin) indicating a lack presented on osteoclastogenesis, and thus leading to increased bone resorption (Freitag et al., 2023; Schluessel et al., 2022; Inoue et al., 2021). Therefore, after briefly exposing some of the biological activity involved in whether an implant system is accepted or rejected, biomarkers are crucial in understanding and improving the healing process of dental implants, particularly regarding osseointegration (Baseri et al., 2020; Rahmati et al., 2020). The success of this integration relies on various biological and biochemical factors, many of which can be tracked using specific biomarkers. The existing literature describes a series of modification techniques to increase the promotion and activity of biomarkers through molecular techniques, coating experiments, and the employment of new biomaterials (Bandyopadhyay et al., 2023; Mateu-Sanz et al., 2024). This systematic review aims to provide a specific targeted overview of new methodologies and findings in methodological advancements for analyzing gene expression and biomarkers that promote osseointegration in dental implants. All research articles published from January 2020 to May 2025 were included for the systematic review.

## 2 Materials and methods

The diversification in the study of gene expression for dental prostheses and prostheses, in general, has expanded to multidisciplinary fields, looking forward to optimizing the osseointegration process and increasing the short-, mid-and long-term success rate. Some of the study fields focused on molecular activity rely on general biomarkers for acceptance and failure of dental implants, specific cases for gene activity (pathologic conditions), dental implant surface coatings, biomaterials, and additive manufacturing materials to maximize the effects of growth factors. Hence, the study approach will be targeted to highlight the findings and contributions in enhancing molecular activity promoting dental implant success rates from multidisciplinary perspectives, in order to respond the PICO research question of “In patients (human) or preclinical models (*in vivo*, *in vitro*) requiring or simulating dental implantation (P), what are the specific molecular mechanisms towards osseointegration (I) and how they are being optimized with omics and technological trends (C), for the identification of key pro-osteogenic and pro-inflammatory biomarkers and pathways? (O)”. A comprehensive literature search relied on the guidelines presented by the Preferred Reporting Items for Systematic Reviews (PRISMA) methodology to establish a thorough and replicable review process. The Meta-analysis was conducted across three central recognized indexed databases; MEDLINE (via PubMed), Scopus and Web of Science, and included all relevant articles from January 2020 to May 2025. The search strategy was specifically designed to identify studies focused on biomarkers and specific gene patterns towards osseointegration, being positive or negative predictive outcomes. To achieve this, a precise search string was developed that combined keywords for the sample type with keywords. The string utilized Boolean operators to link these concepts were: (Dental Implants OR Osseointegration OR dental implant OR osseointegration) AND (Gene Expression OR Transcriptome OR Biomarkers OR gene expression OR molecular signature OR biomarker OR cytokine OR growth factor OR RUNX2 OR osteocalcin OR osteopontin OR RANKL OR OPG OR TNF OR interleukin) AND (osseointegration OR dental implants OR dentistry OR modified surface OR control surface) AND (2020/01/01: 2025/05/31). Table 1 presents the different text strings in natural language and also reports the Boolean operators for the terms employed while conducting the database search. After completing this database search, the retrieved articles were advanced to a selection phase, where they were evaluated for data extraction based on pre-established inclusion criteria; the criteria in this assessment were restricted to original research; the evaluation focused on data derived from observational studies and experimental trials. The evaluation focused on original data from observational studies and experimental trials. Narrative and systematic reviews identified during the search process were excluded from the primary synthesis but were retained for contextual discussion and are summarized separately. These studies needed to focus on reporting the gene activity outcomes.

**TABLE 1:** String natural language methodology on databases search.

Research Combination	Natural language string search methodology on databases (MEDLINE (via PubMed), Scopus and Web of Science
(Biomarkers OR Gene Expression OR Coatings OR Advances OR Trends) AND (Osseointegration OR Dental Implants OR Dentistry) AND (Dental Implants OR Osseointegration OR dental implant OR osseointegration) AND (Gene Expression OR Transcriptome OR Biomarkers OR gene expression OR molecular signature OR biomarker OR cytokine OR growth factor)	((“Biomarkers” OR “biomarker” [All Fields] OR “biomarkers” [All Fields] OR “Gene Expression” OR “gene expression” [All Fields] OR “Coated Materials, Biocompatible” OR “coating” [All Fields] OR “coatings” [All Fields] OR “advances” [All Fields] OR “trends” [All Fields])) AND ((“Osseointegration” OR “osseointegration” [All Fields] OR “Dental Implants” OR “dental implant” [All Fields] OR “dental implants” [All Fields] OR “Dentistry” OR “dentistry” [All Fields] AND (“2020/01/01 : 2025/05/31”) AND (English))
(Biomarkers OR Gene Expression) AND (Osseointegration OR Dental Implants) AND (Dental Implants OR Osseointegration OR dental implant OR osseointegration) AND (Gene Expression OR Transcriptome OR Biomarkers OR gene expression OR molecular signature OR biomarker OR cytokine OR growth factor OR interleukin)	((“Biomarkers” OR “biomarker” [All Fields] OR “biomarkers” [All Fields] OR “Gene Expression” OR “gene expression” [All Fields])) AND ((“Osseointegration” OR “osseointegration” [All Fields] OR “Dental Implants” OR “dental implant” [All Fields] OR “dental implants” [All Fields] AND (“2020/01/01 : 2025/05/31”) AND (English))
(Coatings OR Advances OR Trends) AND (Dental Implants OR Dentistry) AND (Dental Implants OR Osseointegration OR dental implant OR osseointegration) AND (Gene Expression OR Transcriptome OR Biomarkers OR gene expression OR molecular signature OR biomarker OR cytokine OR growth factor OR interleukin)	((“Coated Materials, Biocompatible” OR “coating” [All Fields] OR “coatings” [All Fields] OR “advances” [All Fields] OR “trends” [All Fields])) AND ((“Dental Implants” OR “dental implant” [All Fields] OR “dental implants” [All Fields] OR “Dentistry” OR “dentistry” [All Fields] AND (“2020/01/01 : 2025/05/31”) AND (English))

### 2.1 Eligibility criteria

The review was structured according to the following PICO (Population, Intervention, Comparison, Outcome) to respond the research question:

Population (P): *In vitro* (cell lines, organoids), *in vivo* (animal models), and human studies related to dental implant osseointegration.
Intervention (I): Assessment of gene expression patterns, biomarkers, or molecular signaling pathways for specific molecular mechanisms towards osseointegration.
Comparison (C): A comparison between baseline or traditional understandings of osseointegration and the effects of new technologies (like surface modifications).
Outcome (O): Markers of successful osseointegration (upregulation of osteogenic genes like *RUNX2*) or implant failure (upregulation of inflammatory cytokines like *IL-6*, imbalanced *RANKL/OPG ratio*).

#### 2.1.1 Inclusion criteria

Primary research studies (*in vitro*, *in vivo* animal, prospective/retrospective clinical).
Studies investigating the expression of at least one gene or protein biomarker relevant to osseointegration.
Articles published in English from January 2020 to May 2025.

#### 2.1.2 Exclusion criteria

Study designs such as narrative reviews, systematic reviews, case reports, commentaries, and simulation-only studies.
Studies not focused on the molecular or genetic aspects of osseointegration.

### 2.2 Quantitative synthesis

For the meta-analysis, a subset of the included primary studies was selected based on a stricter set of criteria to ensure homogeneity. Studies had to be an *in vitro* experimental study, compare an experimental implant surface modification against a control or traditional surface, measure the expression of a standard key osteogenic biomarker (*RUNX2*, *OCN*, *ALPL*) and report the outcome as a continuous variable, providing the mean, standard deviation (SD), and sample size (n) for both the experimental and control groups. Due to the included studies measuring gene expression using different assays and potentially different scales or units, a direct comparison of the raw mean values is inappropriate. To address this, the Standardized Mean Difference (SMD) was calculated as the effect size for each study. The SMD exposes the size of the treatment effect in each study relative to the variability observed in that study, effectively converting all results to a uniform dimensionless scale that can be meaningfully compared and combined. The individual SMDs from each study were combined to calculate a single overall effect estimate using a random-effects model. This model is the standard approach for medical and biological research as it assumes the actual treatment effect may differ between studies due to variations in protocols, materials, or other conditions. The random-effects model accounts for both the within-study variance (sampling error) and the between-study variance (heterogeneity), developing a more conservative and robust estimate of the overall effect. Hence, to evaluate the degree of variation between the results of the included studies, statistical heterogeneity was assessed. The I^2^ statistic was calculated to quantify this variation. A high I^2^ value (>60%) indicates that there is substantial heterogeneity, suggesting that the studies are not all estimating the same effect. All statistical analyses were performed using appropriate statistical digital tools (Cochrane tools).

### 2.3 Risk of bias assessment

The methodological quality and Risk of Bias (RoB) of the presented literature were systematically assessed to determine the reliability of the evidence base, with a particular focus on the articles providing foundational evidence for the key molecular signatures of osseointegration, for all other primary studies including non-randomized preclinical *in vivo* animal trials, *in vitro* laboratory experiments, and prospective or retrospective clinical studies the Risk of Bias in Non-randomized Studies of Interventions (ROBINS-I) tool was applied. This tool assesses bias across seven key domains; confounding, participant selection, intervention classification, deviations from intended interventions, missing data, outcome measurement, and selection of the reported result, as well as the overall Risk of Bias. This structured assessment was applied across the three thematic groups central to this review, the foundational studies on pro-osseointegration biomarkers, the key studies on inflammatory and failure-associated pathways, and the seminal reviews and studies on key signaling pathways. The results of these assessments informed the overall Risk of Bias judgments for each study and contributed directly to the final certainty ratings in the GRADE summary of findings table.

## 3 Results

**FIGURE 1:**
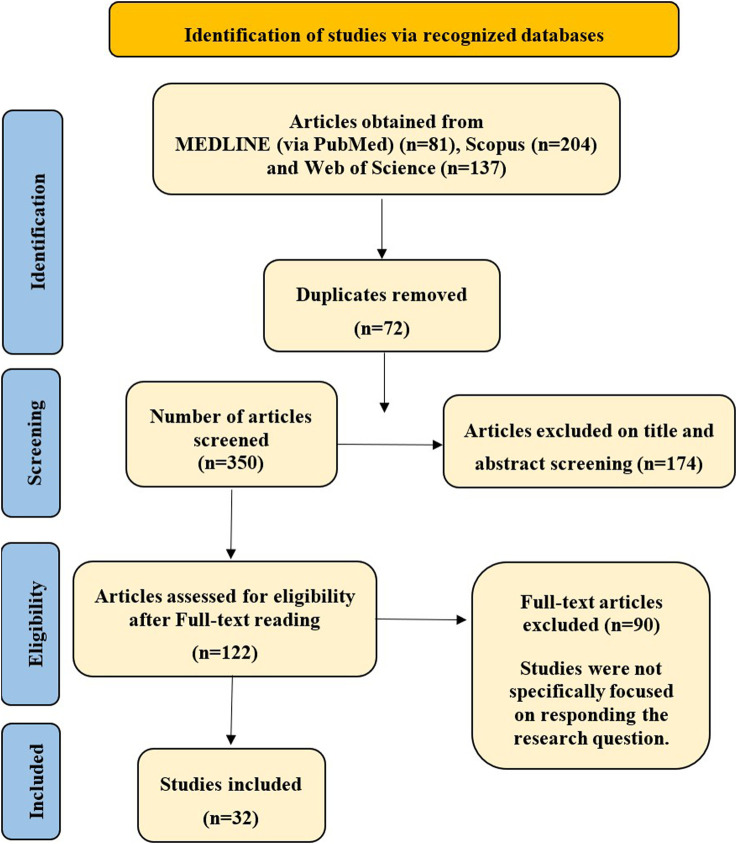
Search flow chart of the literature review.

The systematic MEDLINE (via PubMed), Scopus and Web of Science database search identified 341 potential records. Once exclusion and deduplication for irrelevant references were performed, 296 unique articles were retained for further evaluation. The first level of screening, which involved an independent review of titles and abstracts, excluded 174 articles, leaving 122 studies for a more detailed assessment. Consequently, in order to keep refining the literature search, the full texts of these 122 articles were thoroughly examined. Further in-depth assessment led to the exclusion of 90 additional articles, which were not aligned with the specific inclusion criteria established for this systematic review. Thus, the final compilation of literature in this meta-analysis consists of 32 studies. The PRISMA flow chart visually represents the complete, step-by-step process of study identification and selection (Figure 1).

### 3.1 Qualitative synthesis

The 32 primary studies included in the qualitative synthesis are detailed in Table 2. The evidence base comprises a mix of *in vitro* cellular studies, *in vivo* animal models, and human clinical trials. To ensure methodological clarity and prevent the conflation of different evidence tiers, the findings from these studies are presented below, stratified by study type. In addition, a new table has been created to document the studies excluded at the full-text stage, along with the primary reason for exclusion for each (Supplementary Table S1).

**TABLE 2:** Characteristics of included primary studies.

Study Design	Model (Species/Tissue)	Key Biomarkers/Genes Investigated	Assay Method	Implant Surface/Condition	References
*In vitro* & *in vivo* experimental study	*In vitro*: Human mesenchymal stem cells (*hMSCs*). *In vivo*: Six male beagle dogs (mandibular alveolar ridge)	*RUNX2*, *OCN*, *OPN*, *ALPL*, *COL1A1*. Also, *ALP* activity, matrix mineralization	RT-qPCR, Western blot, *ALP* staining, Alizarin Red S staining, immunofluorescence, micro-CT, histomorphometry	Sandblasted, large-grit, acid-etched (SLA) Titanium (ST) coated with reduced Graphene Oxide (rGO). Compared against ST control and rhBMP-2 coated/treated ST.	Shin et al. (2022)
Clinical research and case reports	Human patients with Titanium (Ti-Impl) and ceramic (Cer-Impl) dental implants. Jawbone tissue samples	C-C motif chemokine 5 (*CCL5*, also known as RANTES)	Trans-alveolar ultrasonography (TAU), Titanium stimulation test (Ti-Stim), multiplex analysis of cytokine expression	Titanium implants (Ti-Impl) vs. Ceramic implants (Cer-Impl) in areas with Bone Marrow Defects of the Jaw (BMDJ)	Lechner et al. (2024)
Bioinformatics analysis and *in vivo* animal study	In silico: RNA-seq datasets (GSE76364, GSE76365). *In vivo*: Type II Diabetes Mellitus (T2DM) rats and normal rats with bone implants	Differentially expressed mRNAs and miRNAs, including *Smpd3*, *Itga10*, and *rno-mir-207*	Bioinformatic analysis (R package limma, KEGG, GO), miRNA-mRNA interaction analysis (miRWalk 2.0), RNA sequencing	Not applicable (bioinformatics); bone implants in T2DM vs. normal rat models	Wang et al. (2021a)
*In vitro* co-culture experimental study	Human periprosthetic fibroblast-like cells (PPFs) from failed dental and orthopaedic implants co-cultured with peripheral blood mononuclear cells (PBMCs)	*OPG*, *RANKL*, *TRAP*, *TNF-α*	RNA-sequencing, *TRAP* staining, resorption assay on dentin discs	Not applicable (cell culture study). Compared monolayer vs. transwell culture systems to mimic *in vivo* conditions	Schluessel et al. (2022)
Retrospective clinical study	193 human patients (72 on oral anticoagulants, 121 controls) undergoing dental implantation	Bleeding events	Retrospective chart review	Not specified. Study focused on patient medication status (oral anticoagulants vs. control)	Manor et al. (2021)
Randomized clinical trial	28 human subjects with single dental implants exhibiting soft tissue dehiscence. Peri-implant crevicular fluid (PICF) collected	Angiogenin (*ANG*), Fibroblast growth factor-2 (*FGF-2*), Platelet-derived growth factor (*PDGF*), Tissue inhibitor of metalloproteinases-2 (*TIMP-2*), Vascular endothelial growth factor (*VEGF*)	ELISA, clinical measurements, Doppler ultrasonography	Implants treated with connective tissue graft (CTG) via either coronally advanced flap (CAF) or tunnel technique (TUN)	Tavelli et al. (2024)
Narrative review (describes primary healing dynamics)	Human palatal tissue (donor site for soft-tissue grafts)	Genes implicated in palatal wound healing, growth factors	Histology, clinical observation	Palatal wound healing after free gingival graft (FGG) or connective tissue graft (CTG) harvesting	Tavelli et al. (2023)
*In vitro* & *in vivo* clinical study	*In vitro*: Human bone marrow stem cells (*hBMSCs*), human microvascular endothelial cells (*HMEC-1*). *In vivo*: 10 human patients (split-mouth design)	*VEGF*, *TGF-β1*, *BMP-2*, *MMP-2*, *MMP-9*, *RUNX2*, *COL1a1*, *OCN*.	SEM, ELISA, Alizarin Red S staining, Real-Time PCR, clinical evaluation (VAS, radiography, probing)	Titanium implants permeated with Concentrated Growth Factor (CGF) vs. traditional (uncoated) implants	Palermo et al. (2022)
*In vitro* experimental study	Macrophages and gingival fibroblasts	Pro-inflammatory cytokines (*TNF-α*, *IL-6*) and pro-regenerative cytokines (*IL-4*, *IL-10*)	Electrophoretic deposition (EPD), ELISA, cell adhesion/proliferation assays, collagen secretion assay	Titanium substrates with zinc-containing chitosan/gelatin (Zn/CS/Gel) coatings	Han et al. (2025a)
*In vivo* experimental animal study	Six beagle dogs; implants placed in hemimandibles	Bone-to-Implant Contact (BIC), Peri-implant Bone Fraction (BF), Interthread Bone (IB)	Histomorphometric analysis, Backscattering Scanning Electron Microscopy (BS-SEM)	CP-Ti dental implants biofunctionalized with *TGF-β1* inhibitor peptides (P17 and P144) vs. untreated control implants	Cirera et al. (2020)
*In vitro* & *in silico* experimental study	*In vitro*: Mouse osteoblasts (*MC3T3-E1* cell line). *In silico*: Finite Element Analysis (FEA)	Osteoblast proliferation	Cell culture contrast experiment, *in vitro* fatigue testing, FEA.	3D printed complex porous Titanium implants filled with biodegradable magnesium alloy	Zhang et al. (2021a)
Prospective clinical trial	10 human patients with atrophic anterior maxillae. Bone biopsies taken from graft area	Osteocalcin (*OCN*), Runt-related transcription factor 2 (*RUNX2*)	Immunohistochemistry (IHC), histomorphometry	Reconstructions using particulate bone xenografts, either alone (Control) or combined with autogenous bone marrow aspirate concentrate (BMAC) (Test)	Hergem et al. (2022)
*In vivo* experimental animal study	26 male rabbits (*Oryctolagus cuniculus*) with surgically created tibial bone defects	*RUNX2*, *BSP*, *OPN*, *OCN*, *OPG*, *RANKL*.	RT-PCR, Immunohistochemistry (IHC), Micro-CT analysis	Porous surface (hydrophobic) vs. Porous-hydrophilic surface. Both tested with defects filled with either blood clot or a biphasic HA/TCP bone substitute	dos Santos Trento et al. (2020)
*In vitro* experimental study	Mouse preosteoblast cells (MC3T3-E1)	*ALPL*, *OCN*, *RUNX2*, *OPN*, β-catenin, Integrin β1, Cyclin D1	RT-qPCR, SEM, XRD, GD-OES, wettability test, Alizarin Red staining, *ALPL* activity assay	Selective Laser Melting (SLM) fabricated Titanium (SLM-Ti) with high roughness, treated with mixed acid and heat (MAH)	Le et al. (2021)
*In vitro* & *in vivo* experimental study	*In vitro*: Human embryonic palatal mesenchymal pre-osteoblasts. *In vivo*: Beagle dogs	*RUNX2*, *OPN*, *OCN*, Alkaline Phosphatase (*ALPL*) activity	Real-time PCR, Western blot, ELISA, Histology, BIC ratio measurement	Titanium discs and implants coated with flame-sprayed Hydroxyapatite (HA) doped with Strontium (Sr) and Magnesium (Mg) (5Sr5Mg-HA)	Wang et al. (2020)
*In vitro* proteomic and experimental study	Human osteoblasts (HOb)	Proteome-wide analysis (2544 proteins); *COLI*, *RUNX2*, *SP7*, *CTNNB1*, *CAD13*, *IGF2*, *MAPK2*, *mTOR*.	Nanoflow liquid chromatography tandem mass spectrometry (nLC-MS/MS), RT-qPCR, cell proliferation and mineralization assays	Sandblasted acid-etched (SAE) Titanium implants vs. cells cultured without Ti	Romero-Gavilán et al. (2023)
Prospective clinical study	38 human patients with implant-supported crowns. Gingival crevicular fluid (GCF) collected	*IL-6*, *TNF-α*, *TGF-α*, *OCN*.	ELISA.	Implants with crowns retained by adhesive vs. a modified adhesive technique	Wang et al. (2021b)
*In vitro* experimental study	Human osteoblasts and fibroblasts	*IL-1β*, *OPN*, *COL1A1*, *IL-8*, *ALPL* activity	Cell viability assays, ELISA.	Yttria-stabilized Zirconia (YSZ) discs with different surface textures, conventional milling, Nd:YAG laser, and sandblasted/acid-etched (control)	da Cruz et al. (2022)
*In vitro* experimental study	Rat bone marrow-derived osteoblasts	Cell attachment, proliferation, *ALPL* activity, mineralized matrix formation	*WST-1* colorimetric assay, fluorescence microscopy, Alizarin Red staining	Sandblasted vs. acid-etched Ti implants; hydrophilic vs. hydrophobic surfaces; culture with/without N-acetylcysteine (NAC). All tested in a novel 3D dynamic culture system	Komatsu et al. (2025)
*In vitro* & *in vivo* experimental study	*In vivo*: Rabbits. *In vitro*: Bone marrow mesenchymal stem cells (*BMSCs*), human immortalized keratinocytes	Osteogenic differentiation markers, bacterial adhesion	SEM, contact angle, RT-qPCR, Alizarin Red S staining, live/dead staining, radiological, fluorescent, and histomorphometric analyses	Self-glazed Zirconia (SZr) with micro/nano-roughened surface vs. sandblasting large-grit acid-etching treated Titanium (Ti-SLA)	Zhang et al. (2023a)
*Ex vivo* human implant retrieval study	20 retrieved human Titanium dental implants	Bone Collagen Fibre Orientation (BCFO)	Scanning Electron Microscopy (SEM), Circularly Polarized Light Microscopy (Birefringence Analysis)	Six groups (A-F) of Titanium implants with different thread designs (V-shaped, concave shaped)	Valente et al. (2021)
*In vivo* experimental animal study	Six beagle dogs; implants placed in radii	Bone to Implant Contact (%BIC), Bone Area Fraction Occupancy (%BAFO), Insertion Torque	Histologic and histomorphometric analyses	Type V Ti alloy implants with two different thread profiles	Benalcázar-Jalkh et al. (2023)
Prospective clinical study	60 healthy human patients. Peri-implant sulcular fluid (PISF) collected	*OPN*, *OCN*, *ALPL*, *OPG*, Nitric oxide (NO)	ELISA.	Conical-shaped implants (CI) vs. Hexagonal implants (HI)	Bayandurov et al. (2024)
*In silico* computational modeling study	Finite element model of an immediately loaded dental implant	Tissue differentiation (bone, soft tissue), bone density, implant micromotion	Mechano-regulatory cellular differentiation model (poroelastic modeling for healing, density adaptation for remodeling)	Not applicable (simulation study). Varied initial implant micromotion	Irandoust and Müftü (2020)
Retrospective clinical study	46 human patients (64 bone-level implants)	Marginal bone level (MBL), periodontal probing depth	Radiographic analysis, clinical measurements	Two-stage bone-level dental implants after 1–1.5 years of functional loading	Maghsoudi et al. (2022)
*In vitro* & *in vivo* experimental study	*In vivo*: Minipigs. *In vitro*: Material characterization	Bone-to-Implant Contact (BIC)	Roughness/microhardness testing, residual stress analysis, fatigue testing, histomorphometry	Titanium implants sandblasted with Al_2_O_3_ vs. TiO_2_, followed by acid etching	Gi et al. (2022)
*In vivo* experimental animal study	Six healthy New Zealand rabbits; implants in femur	Bone mineral density (BMD), bone mineral content (BMC), bone volume fraction (BV/TV), collagen secretion, BIC.	Micro-CT, Hematoxylin and Eosin (H&E) staining, Masson’s trichrome (TRI) staining	Nanostructured CP-Ti (via caliber rolling) vs. standard CP-Ti, with either SLA or SLActive surface modification	Sadrkhah et al. (2023)
*In vivo* experimental animal study	21 male Sprague-Dawley rats with silk-ligature induced peri-implantitis	Crestal bone level, osteoclasts, inflammatory cells, *IL-6*	Histology (H&E staining), TRAP staining, Immunohistochemistry (IHC)	Titanium implants. Test group received topical application of a *p65-TMD-*linked *PTD* (*NF-κB* inhibitor)	Jung et al. (2021)
Prospective clinical study	78 human patients with single posterior mandibular implants. PICF collected	*IL-1β*, *TNF-α*, *IL-10*	ELISA.	Three implant microgeometries: sandblasted acid-etched (SLA), SLActive, and anodized (TiUnite)	Gnanajothi and Rajasekar (2024)
*In vitro* & *in vivo* experimental study	*In vivo*: Rats with LPS-induced peri-implantitis. *In vitro*: Co-culture of gingival fibroblasts and osteoclasts/osteoblast	Proinflammatory cytokines (*TNF*, *IL-1β*, *IL-6*), osteoclast numbers, *TLR4*, *NF-κB* activation	Histology, cytokine analysis, Western blot	Titanium implants. Test group received daily administration of melatonin	Wu et al. (2021)
*In vitro* experimental study	Human osteoblast-like cells (*MG-63*)	*NF-κB* pathway activation (phospho-p65), MHC class I and II proteins	Immunoblot, immunofluorescence, MTT assay, flow cytometry	Polished Titanium vs. polished zirconia discs	Mukaddam et al. (2025)
*In vitro* experimental study	Human osteoblast-like cells (*Saos-2*)	*RUNX2*, *SP7/OSX*, *ALPL*, *COL1A1*, *SPP1/OPN*, *BGLAP/OCN*.	RT-qPCR, cell viability assays (MTT), *ALPL* activity assay	Differently treated Titanium surfaces for subperiosteal implants: machined, sandblasted, acid-etched, and double acid-etched	Roy et al. (2024)

#### 3.1.1 Synthesis of principal from *in vitro* studies

*In vitro* analyses provide a controlled environment for evaluating the direct cellular and molecular responses to specific biomaterials and conditions since they are targeted to eliminate the systemic complexities of a living organism. Validating bioactive surface coatings using the power of *in vitro* models to test novel surfaces was revealed by Shin et al. (2022), culturing human mesenchymal stem cells (hMSCs) on a Titanium surface coated with reduced Graphene Oxide (rGO); the researchers could directly measure the cellular response to the material. This model found significant upregulation of a suite of crucial osteogenic genes, including the master regulator *RUNX2* and late-stage markers like *OCN*, *OPN*, *ALPL*, and *COL1A1*. Likewise, Palermo et al. (2022) reported the efficacy of autologous biologics since the study utilized human bone marrow stem cells (*hBMSCs*) to validate the bioactivity of Concentrated Growth Factor (*CGF*), an autologous biologic. Furthermore, it is relevant to note the study of an *in vitro* co-culture model to deconstruct the mechanisms of implant failure by Schluessel et al. (2022). The researchers could observe the complex cellular crosstalk that occurs during peri-implantitis by using periprosthetic fibroblast-like cells harvested from actual failed implants and co-culturing them with immune cells (PBMCs).

#### 3.1.2 Synthesis of principal from *in vivo* animal studies

*In vivo* animal models are a crucial next step in translational research, enabling the evaluation of molecular findings within a complex, living biological system that incorporates a functional immune response, vascularization, and mechanical loading. The *in vivo* study by Shin et al. (2022) provided essential preclinical validation for its *in vitro* findings. The rGO-coated implants in a beagle dog model, a large animal model with high relevance to human dentistry due to its mandibular bone structure and healing patterns, the researchers could assess the material’s performance in a real biological environment. To robust the systemically compromised models, the unique strength of animal models to study systemic disease was leveraged by Wang et al. (2021b). This study combined a bioinformatic approach with an *in vivo* model of Type II Diabetes Mellitus (T2DM) in rats. This model is invaluable for understanding why osseointegration can be impaired in patients with diabetes. The insight gained was the identification of specific differentially expressed mRNAs and miRNAs (including Smpd3, Itga10, and rno-mir-207) that are directly implicated in the altered bone healing pathways associated with diabetes.

#### 3.1.3 Synthesis of principal findings from human clinical studies

Identifying clinically relevant biomarkers of failure was supported by direct clinical evidence from analyzing jawbone tissue samples from patients with both titanium and ceramic implants. It has also been observed that the clinical portion of the study by Palermo et al. (2022), which tested the CGF-permeated implants, provided the ultimate translational insight. The CGF-coated implants, which showed promise at the cellular level *in vitro*, led to demonstrably positive clinical and radiographic outcomes in human patients. The assessment of soft tissue healing and patient factors was reported in a randomized clinical trial by Tavelli et al. (2024), focused on the crucial aspect of peri-implant soft tissue healing. By collecting peri-implant crevicular fluid (PICF), a non-invasive method for sampling the biomolecular environment around an implant, the study measured key angiogenic and growth factors, including *ANG*, *FGF-2*, and *VEGF*. According to the results were that different soft tissue grafting techniques directly modulated the levels of these biomarkers related to healing. In this regard, the study by Gnanajothi and Rajasekar (2024) indicated a real-world context by examining how patient-related factors; such as the use of oral anticoagulants and impact clinical outcomes, when considering the whole patient in treatment planning.

### 3.2 Study selection for meta-analysis

**FIGURE 2:**
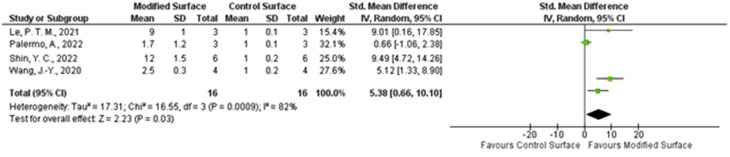
Foster plot of modified and control surfaces for *RUNX2*.

From the 32 studies included in the systematic review, a smaller, homogenous subgroup was identified as suitable for quantitative meta-analysis. The most frequently reported and consistently measured outcome suitable for pooling was the relative gene expression of the master osteogenic transcription factor, *RUNX2*, in *in vitro* studies comparing a modified Titanium surface to a control surface. Four group studies met all the inclusion criteria for this meta-analysis (Shin et al., 2022; Palermo et al., 2022; Le et al., 2021; Wang et al., 2020). The data extracted from the four selected studies for the meta-analysis of *RUNX2* gene expression are presented in Figure 2. A random and effects model was employed to pool the Standardized Mean Difference (SMD) from each study in RevMan software. The results of the random-effects are summarized in the forest plot (Figure 2). The analysis revealed that modified Titanium surfaces led to a significant increase in *RUNX2* gene expression compared to control surfaces, the pooled Standardized Mean Difference (SMD) was 2.4 (95% CI) and the assessment of heterogeneity showed an I^2^ statistic of 82% (p = 0.0009). This high value indicates that there is substantial heterogeneity among the studies, meaning that approximately 82% of the variability in the observed effect sizes is due to genuine differences between the studies (such as the specific surface modification used or the experimental protocols) rather than sampling error. While the overall direction of the effect is consistently positive, its magnitude varies significantly across the pooled investigations.

The certainty of the evidence for the key molecular findings was assessed using the GRADE framework. Four primary outcomes were evaluated: the effect of modified surfaces on pro-osteogenic gene expression, the association of pro-inflammatory cytokines with implant failure, the association of the *RANKL/OPG* ratio with implant failure, and the modulation of key signaling pathways by implant surfaces. The evidence for all outcomes was rated as Low or Very Low certainty. The detailed assessment, including the rationale for downgrading the certainty of evidence for each outcome, is presented in the Summary of Findings table (Table 3).

**TABLE 3:** GRADE summary of findings for key biomarkers and pathways.

Outcome	No. of Participants (Studies)	Certainty of the Evidence (GRADE)	Summary of Findings
Upregulation of Pro-Osteogenic Markers (*RUNX2*) by Modified Surfaces	Not applicable (13 primary studies, including 6 *in vitro* studies in meta-analysis)	LOW	Serious concerns due to the inclusion of non-randomized preclinical studies with moderate to serious risk of bias Substantial statistical heterogeneity in the meta-analysis (I^2^ = 82%) and clinical heterogeneity across different models and surface types The evidence is predominantly from *in vitro* and animal models, which are indirect surrogates for human clinical outcomes
Association of Pro-Inflammatory Cytokines (*IL-1β*, *IL-6*, *TNF-α*) with Implant Failure	Not applicable (11 primary studies: clinical, animal, *in vitro*)	LOW	Serious concerns, particularly from the non-randomized clinical studies which have a high risk of confounding Significant clinical heterogeneity in study designs, patient populations, and the specific panels of cytokines measured Mechanistic evidence is primarily derived from preclinical models, which is indirect evidence for human peri-implantitis The evidence was upgraded one level due to the very large effect sizes reported in some studies (20-fold increase in *CCL5*)
Association of Increased *RANKL/OPG* Ratio with Implant Failure	Not applicable (3 primary studies: clinical, animal, *in vitro*)	LOW	The evidence is sparse and derived from different models (*in vitro* co-culture, animal models, clinical fluid analysis), making it difficult to confirm consistency The evidence is entirely from preclinical models or surrogate clinical markers (PISF), which is indirect The evidence was upgraded one level due to the large magnitude of the effect observed (20-fold decrease in *OPG*)
Modulation of Key Signaling Pathways (*NF-κB, Wnt*) by Implant Surfaces	Not applicable (7 primary studies: *in vitro*, animal)	VERY LOW	Serious concerns due to the inclusion of non-randomized preclinical studies, some with serious risk of confounding (T2DM model) High clinical heterogeneity, as studies investigate different pathways (*NF-κB, Wnt, PI3K-Akt, Hippo-YAP*) in response to varied stimuli (surfaces, ions, disease states) The evidence is entirely from preclinical models, which are indirect surrogates for complex signaling in human patients

### 3.3 Specific dental implant success and failure gene activity and signaling pathways

**FIGURE 3:**
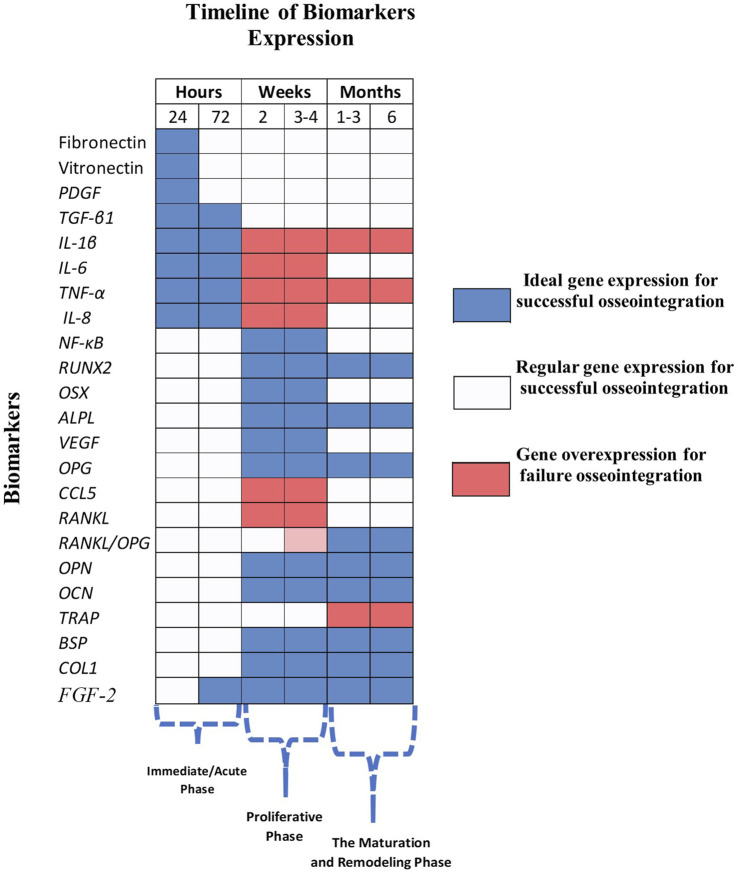
Timeline of biomarker expression heatmap.

During a dental implant intervention, a biological cascade of intercellular signaling is triggered, promoting the success or rejection of the implant system, which is influenced by genetic pathways. This process initially occurs once the implant is fully covered in blood (Hemostasis); this natural process tends to form blood clots along the biological tissue between the implant system surface (Manor et al., 2021; La Monaca et al., 2023). It has been well-documented that a fibrin scaffold, including the first proteins, is expressed within minutes. The initial proteins presented are Fibrinogen (*FGN*), albumin, and Fibronectin (*FN*), which remain crucial for adequate clot formation (Liu et al., 2021; Kuchinka et al., 2021). Blood platelets close blood vessels ruptures from the intervention; likewise, this event generates a series of signaling for cell communication promoting cytokines or growth factors platelet derived growth factors (*PDGF*), fibroblast growth factor (*FGF*), angiogenin (*ANG*), endothelial growth factor (*VEGF*) and transforming growth factor-beta (*TGF-β*), which initially aid in healing the wound (Tavelli et al., 2024; Tavelli et al., 2023; Kligman et al., 2021), creating a temporary matrix that covers the wound and allowing the growth factors to adhere the implant system surface. Recent studies have demonstrated that, within the first few minutes after the implant system is placed, the inflammatory response is crucial in determining the wound healing rate and, thus, the primary stability of the implant system. In this initial stage, the evaluation of gingival crevicular fluid (GCF) and peri-implant crevicular fluid (PICF) has revealed the presence of growth factors, including angiostatin, *PDGF-BB*, *VEGF*, *FGF-2*, and interleukin-8 (*IL-8*) (Tavelli et al., 2024; Palermo et al., 2022). During the initial post-implantation days, the stimulation of growth factors is widely presented. The cascade reaction to the wound healing takes relevance in the release of macrophages, keratinocytes and fibroblasts to promote genetic expression of most of the genes expressed in the first hours post-implantation as well as Tumor necrosis factor (*TNFα*), Transforming growth factor (*TGFα*) and Epidermal growth factor (*EGF*); leading to scar tissue formation (Baker et al., 2024; Tavelli et al., 2024; Han et al., 2025a; Cirera et al., 2020). The transcription factors along the extracellular matrix trigger osteoblast proliferation, promoting the increment in the bone regeneration process through the construction of fibrin scaffolds (Zhang X. et al., 2021; Matsuura et al., 2024). Several investigations have reported the role of Runt-related transcription factor 2 (*RUNX2*) as a primary regulator of osteoblastogenesis and a modulator of calcification inhibitor concentrations through Alkaline Phosphatase (*ALPL*) during the early stages of implant system differentiation (Hergem et al., 2022). In addition, increased expression of *RUNX2* was observed, followed by Collagen 1 (*COL1*), *ALPL*, Bone sialoprotein (*BSP*), and Osteocalcin (*OCN*) (dos Santos Trento et al., 2020; Le et al., 2021; Wang et al., 2020). Specifically, *COL1* has been the subject of study to determine its proteomic production, as it comprises around 90% of the total collagen in bone tissue. *COL1* acts as a functional coating on the surface of dental implants, mimicking the natural interface and promoting osteoblast adhesion, which ultimately enhances bone mineralization and osseointegration. It is essential to emphasize the role of *COL1*, even in compromised bone, as demonstrated in recent research using osteopenic rat animal models where it effectively promotes implant osseointegration *in vivo* (Wang Z. et al., 2023). The presence of *COL1* is associated with bone regeneration from bone marrow cells and an increase in the biological activity of related differentiation genes, such as *OCN*, *BSP*, and *ALPL* (Romero-Gavilán et al., 2023; Han et al., 2025b). Increased *OCN* levels are associated with influencing bone mineralization density and matrix formation (Schluessel et al., 2022; Palermo et al., 2022; Wang et al., 2021b). The Alkaline Phosphatase biomarker has also been documented as the first gene that promotes the calcification of new bone tissue (da Cruz et al., 2022; Komatsu et al., 2025). To visually represent the importance of the evolution of the behavior of gene patterns in early implant placement, a heatmap describing the biomarker-time relationship of some of the main gene expression activity for ideal and failure osteointegration is depicted in Figure 3.

Bone Morphogenetic Protein (BMP) signaling has been reported to regulate the expression of Distal-less homeobox 5 (*DLX5*), which interacts with the enhancer regions of *RUNX2* (Vermeulen et al., 2022). Thus, the *RUNX2* is a vital transcription factor that drives osteogenesis and governs key skeleton-associated genes, such as Osterix (*OSX*) and *OCN*. During initial osteoblast differentiation, a myriad of bone matrix proteins is synthesized. Among these, the expression of Bone Sialoprotein (*BSP)*, along with other bone matrix genes is transcriptionally regulated by *RUNX2* (dos Santos Trento et al., 2020; Zhang W. et al., 2023). The role of *TGF-β* in modulating *RUNX2* expression has been found to promote the complexity of this signaling pathway. Furthermore, SMAD proteins are instrumental in forming complexes with *RUNX2*, effectively guiding its transcriptional activity (Zhang C. et al., 2021). Therefore, during the early weeks of the implant system placement, the biological activity remains primarily in the genes mentioned above; their expression provokes the creation of woven bone in the implant surface, and some osteocytes can be observed in the center of the newly formed bone tissue, while osteoclasts appear on the surface of the resting bone, indicating the resorption of necrotic bone (Valente et al., 2021; Khaohoen et al., 2023). The creation of the new woven bone consists of osteoblasts and osteocyte cells having a non-aligned collagen fiber structure and brittle behavior; as the bony tissue mineralizes, it strengthens and arranges its composition into precisely structured circular lamellar fibers, which now form the structure that can resist functional loading and lead to bone remodeling (Benalcázar-Jalkh et al., 2023; Jose et al., 2023). Bone remodeling occurs in around a one-month time frame when osteoblasts and osteoclasts collaborate to build and resorb bone based on osteoblast Receptor Activator of Nuclear Factor kappa-B Ligand (*RANKL*) signal messenger requirements according to the interfacial tissues through the implant system to have enough stability to withstand functional occlusal loading conditions (Bayandurov et al., 2024; Irandoust and Müftü, 2020; Maghsoudi et al., 2022).

A recurring solid statement in dentistry assessment is that the relationship associated with biomarkers promoting peri-implant conditions is the key factor in dental implant failure cases. In addition, implant failure can be observed under hereditary, metabolic and systemic conditions and mechanobiological overloading due to peri-implant gene expression (Hossain et al., 2023). Primary stability and perioperative contamination are the leading causes of early implant failure. Conversely, peri-implantitis and overloading are the critical factors driving late implant failure (Guo et al., 2021). It has been well-documented that peri-implant diseases inhibit the expression of the main regulatory genes for osteoblast and osteoclast activity; on the contrary, they promote inflammatory-risk conditions and an increment in the periodontal sulcus, thus leading to early implant failure. Loss of surrounding bone tissue within the implant of 0.2 mm periodically (normally monthly) during the first year is a negative pattern for implant rejection in the patient-host system (Jose et al., 2023). Hence, to determine the presence of reliable and readily available sources of gene expression promoting implant failure, the employment of immunoassays in the oral cavity through saliva, GCF, peri-implant crevicular fluid is the most common non-invasive technique utilized in human studies (Asa’ad et al., 2020; Gul et al., 2020; Zhou and Liu, 2023). In this regard, cytokine activity has been widely reported due to elevated levels in the expression of genes, such as interleukin-1β (*IL-1β*), interleukin-6 (*IL-6*), interleukin-10 (*IL-10*), and interleukin-17 (*IL-17*), in the peri-implant crevicular fluid, which is associated with inflammatory responses that can affect osseointegration and implant success. Specifically, *IL-1β* is noted for its significant involvement in bone resorption processes, making it a critical marker for assessing peri-implant health (Lumbikananda et al., 2024; Delucchi et al., 2023). Hence, a myriad of reviews has been comprehensively covered, highlighting the specific focus, novelty or unique contribution, and key molecular mechanisms of failure (gene expression, pathways, biomarkers, polymorphisms). In addition to the primary literature, our search identified five relevant systematic and narrative reviews that provide a broader context on the molecular mechanisms of implant failure. The key findings from these reviews are summarized in Table 4 and are used to inform the discussion of our primary synthesis (Amengual-Penafiel et al., 2021; Choukroun et al., 2024; Lafuente-Ibáñez de Mendoza et al., 2022; Albrektsson et al., 2023; La Monaca et al., 2025).

**TABLE 4:** Summary and comparison of recent reviews exploring molecular mechanisms in dental implant failure process.

Review paper	Specific focus	Novelty or unique contribution	Key molecular mechanisms of failure (gene expression, pathways, biomarkers, polymorphisms)	References
Osteoimmunology drives dental implant osseointegration: A new paradigm for implant dentistry	Role of osteoimmunology in dental implant osseointegration and failure	Proposes osteoimmunology and the Foreign Body Equilibrium (FBE) model as a new paradigm for understanding implant integration and the mechanisms leading to bone loss and failure	Failure associated with an unresolved or reactivated immune-inflammatory response, potentially leading to aseptic loosening or infection High levels of pro-inflammatory cytokines (*TNF-α*, *IL-1β*, *IL-6*, *PGE2*) are associated with peri-implant inflammation and bone loss Accumulation of antigens (metal ions, wear debris, bacterial components) at the implant-tissue interface can act as stress signals, triggering an adverse immune response leading to failure	Amengual-Penafiel et al. (2021)
Bone Formation and Maintenance in Oral Surgery: The Decisive Role of the Immune System—A Narrative Review of Mechanisms and Solutions	Significant connection between the bone and immune systems (osteoimmunology) and the role of oxidative stress in bone health and the success of dental implants and bone grafts	Adopts an osteoimmune perspective to explain the success or failure of dental implants and bone augmentation procedures, aiming to provide a biological explanation for marginal bone loss	Increased production of *RANKL* and pro-inflammatory cytokines like *IL-1β*, *IL-6*, *IL-10*, and *IL-17* The role of T cells in activating osteoclasts, leading to bone resorption Oxidative stress disrupts cellular balance, leading to increased expression of *RANKL* and the activation of interconnected signaling pathways involving Extracellular Receptor Kinase (ERK) and *NF-κB*, pro-inflammatory cytokines *TNFα* and *IL-6*, and matrix-degrading enzymes *MMP-2/8/9*; promoting osteoblast apoptosis and osteoclastogenesis Metabolic conditions, penicillin allergies, local tissue factors, and chronic hypoxia to the exacerbation of inflammation and oxidative stress	Choukroun et al. (2024)
Role of proinflammatory mutations in peri-implantitis: systematic review and meta-analysis	Analise the risk of developing peri-implantitis (PI), synthesized data via meta-analysis to assess the strength of this genetic association	Evaluate the association between proinflammatory gene polymorphisms, specifically in the *IL-1* gene family (IL-1β, *IL-1α*)	Statistically significant association between the C/C genotype of *IL-1β* (−511) and peri-implant bone loss *IL-1β* and *IL-1α* are proinflammatory cytokines implicated in promoting alveolar bone resorption Myeloid differentiation factor-88 (*MyD88*) is mentioned as activating *IL-1β* and *IL-1α* secretion	Lafuente-Ibáñez de Mendoza et al. (2022)
Osteoimmune regulation underlies oral implant osseointegration and its perturbation	Centered on elucidating the osteoimmune interactions that regulate bone modeling and remodeling around dental implants, targeted to pathological failure (peri-implantitis)	Emphasizes the dynamic balance within the osteoimmune system required for health and how its perturbation leads to peri-implant disease, offering a crucial conceptual framework	Activated immune cells, particularly *Th17* lymphocytes and B lymphocytes, serve as significant sources of *RANKL*, directly contributing to osteoclastogenesis Peri-implantitis likely involves upregulation of genes encoding M1 markers (*TNF*, *IL1B*), *Th1/Th17* cytokines (*IFNG*, *IL17A*), *RANKL*, and osteoclast differentiation factors (*NFATc1*) Persistently elevated levels of pro-inflammatory cytokines, including *TNF−α*, *IL−1β*, *IL−6*, and *IL−17*, contribute directly to bone resorption	Albrektsson et al. (2023)
Biomarkers in Peri-Implant Crevicular Fluid of Healthy Implants and Those With Peri-Implant Diseases: A Systematic Review and Meta-Analysis	Comparison of the levels of biological components in peri-implant crevicular fluid (PICF) between healthy implants and those with untreated peri-implant disease (mucositis and peri-implantitis)	Integration and comparison of the different levels of a wide range of 96 different biomarkers assessed in peri-implant crevicular fluid in studies comparing healthy implants with those with mucositis or peri-implantitis, as well as between mucositis and peri-implantitis	Specific role of various molecules in the reported insights of pathophysiology of peri-implant diseases: Proinflammatory cytokines such as *IL-1β*, *IL-6*, *TNF-α*, *IL-17* Osteoclastogenesis-related factors such as *RANK*, *RANKL*, *sRANKL*, *OPG*. Anti-inflammatory cytokines such as *IL-10* Chemokines such as *IL-8*, *MIP-1α/CCL3*, *MIP-3α/CCL-20* Enzymes such as Matrix metallo-proteinase 8 (*MMP-8*), Cathepsin K (*Cat-K*), Aspartate aminotransferase (*AST*)	La Monaca et al. (2025)

### 3.4 Molecular responses to implant surface modifications

Osseointegration enhancement processes are mainly attributed to avoiding failure rates. Dental implant failures, whether occurring early (before functional loading) or late (after loading), are targeted to insufficient or compromised osseointegration. Therefore, understanding and optimizing the biological events that control the formation and maintenance of the Bone-Implant Interface (BIC) remains a critical area of research. Recent technological trends have been applied in dental implantology (Liu et al., 2020).

In implantology literature, it has been presented that the functional surface of a dental implant is more than a passive substrate for bone apposition; it is an active participant in the biological cascade initiated upon implantation. The physicochemical properties of the implant surface, including its topography (roughness at macro, micro, and nanoscales), chemical composition, surface energy, and wettability (hydrophilicity/hydrophobicity), profoundly influence the immediate interactions with biological fluids and subsequent cellular events (Cruz et al., 2022). These surface characteristics generate the adsorption of proteins (quantity, type, and conformation), which mediates the attachment, proliferation, migration, and differentiation of relevant cell activity, most notably Mesenchymal Stem Cells (MSCs) and osteoblast differentiation (Zhou et al., 2020). Consequently, surface modification and coating interfaces have emerged as primary strategies to increment biocompatibility and promote the bio-affinity of implant materials, accelerating and improving the quality of the host bone response (Al-Zubaidi et al., 2020). The modern perspective acknowledges that appropriately modified surfaces are more readily recognized by the host tissue, promoting a more rapid and robust accumulation of bone.

The research literature on removal techniques has primarily focused on altering the surface topography. These physical changes were reported to have significant biological consequences. Specifically, laser-treated surfaces were found to exhibit excellent biocompatibility and an ability to reduce the inflammatory response. Furthermore, laser modifications have been shown to influence cell behavior directly since several *in vitro* studies have reported enhanced adhesion of human osteoblast-like cells and preferential attachment of fibroblasts. This enhanced adhesion was associated with a greater expression of Focal Adhesion Kinase (Gi et al., 2022). Beyond just attachment, laser-textured surfaces were found to promote cell adhesion and proliferation, including the differentiation of MG63 fibroblast-like human osteosarcoma cells. The surface topography was also shown to provide cell contact guidance, resulting in an overall positive interaction between bone cells (Saran et al., 2023).

The literature on additive methods, which involve depositing a layer of material onto the implant surface (Dong et al., 2020), provided evidence that these coatings are designed to actively enhance the biological performance and molecular response at the bone-implant interface (Kligman et al., 2021; Shayeb et al., 2024; Wu et al., 2025). Furthermore, the implementation of bioactive molecules, including growth factors (BMPs, PDGF, VEGF) and ECM components like collagen, was reported to provide crucial osteoinductive signals that enhance cell attachment and differentiation (Gulati et al., 2023; Łosiewicz et al., 2025; Schmalz et al., 2023; Gavinho et al., 2022). Similarly, antibacterial agents, such as Silver (Ag), Cu, and Chitosan, are incorporated to prevent implant-related infections and the associated inflammatory response (Luo et al., 2023). Biodegradable polymers like Polylactic-co-glycolic acid (PLGA) or Polycaprolactone (PCL) are also mentioned in published research as carriers for drug/growth factor delivery or as scaffold components (Mukherjee et al., 2023). Furthermore, coatings incorporating nanoparticles and nanostructures (nano-HA, TiO2 nanotubes, graphene) are used to mimic the natural tissue architecture and directly enhance bioactivity (Teulé-Trull et al., 2025; Tao et al., 2025). The literature also describes a trend toward integrating multiple biological properties onto a single surface (Donos et al., 2023; Miron et al., 2024). His includes combining osteoconductive and osteoinductive elements (like HA, BMPs, Sr, Zn, and Mg) with antibacterial or immunomodulatory components to create a more comprehensive and biologically active bone-implant interface (Donos et al., 2023; Miron et al., 2024). Hence, chemical modifications alter the surface chemistry and often wettability (Vaidya et al., 2024). Creating superior controllable specific surface modifications at micro and nano scale to increase osteogenic cell activity and the interaction of the interface with bony tissue (Komatsu et al., 2024). In this specific regard, fluoride ions can be incorporated specifically to stimulate bone formation, where a primary objective of these chemical modifications is to increase surface hydrophilicity (wettability), as this property has been shown to promote the interaction with biological fluids and cells (Sadrkhah et al., 2023).

Described biological responses can be acquired due to modifications techniques that are commonly employed at the 1–100 nm scale to create bioactive the interfaces (Wen et al., 2023). The primary biological rationale is to mimic the nanoscale architecture of the natural bone ECM, thereby influencing protein adsorption and cellular behavior more effectively. The literature reports that nanoscale surfaces significantly enhance Osterix (*OSX*) expression *in vivo*, suggesting a specific nano-mediated osteoinductive pathway. These surfaces also upregulate *ALPL*, Dentin Matrix Protein 1 (*DMP1*), *BSP*, *OCN*, and Special AT-Rich Sequence-Binding Protein 2 (*SATB2*) expression compared to smooth surfaces, indicating accelerated osteogenesis (Tao et al., 2025; Ma et al., 2023; Li et al., 2023). Moreover, TiO2 nanorod arrays (TNRs) have been shown to upregulate *ALPL*, *RUNX2*, and *OPN* in Periodontal Ligament Stem Cells (PDLSCs). By creating features at the nanometer scale, these surfaces can interact directly with proteins and cellular adhesion molecules (like integrins) to mimic the natural extracellular matrix (Li et al., 2023; Hakim et al., 2024; Shi et al., 2024). Surface modifications at the nanoscale enable more precise control over protein adsorption conformation, such as cell signaling events and subsequent gene expression.

Despite selecting any surface modification method, the translation of physical and chemical surface properties into adequate biological outcomes like enhanced bone formation is mediated by sophisticated initial intracellular signaling pathways (including adhesion, proliferation, differentiation, and apoptosis) (Liu et al., 2021; Albrektsson et al., 2023). Key pathways are responsible for the initial inflammatory response, subsequent osteogenesis, and the continuous process of bone remodeling at the interface. Particularly, the Wingless/Int-1 (*Wnt*) pathway stands as a master regulator of skeletal development and postnatal bone homeostasis, playing a critical role in osteogenesis towards the osteoblastic lineage and regulating the function of mature osteoblasts and osteocytes (Emam and Moussa, 2024). Implant surface characteristics influence *Wnt* signaling. Rough and hydrophilic surfaces, such as SLA and SLActive, appear to promote an M2 macrophage phenotype, and these macrophages can secrete *Wnt* ligands, thereby stimulating osteogenesis locally (Tuikampee et al., 2024). Enhancement of *Wnt* signaling activity has been correlated with accelerated bone healing and improved implant osseointegration, as studies have shown increased *Wnt5A* expression by MSCs on SLA/SLActive surfaces (Jagannathan et al., 2024). Furthermore, The *Wnt/β-catenin* and *Wnt3A* pathways enhance MSC proliferation on smooth Titanium, while *Wnt5A* promotes osteogenic commitment on rough Titanium. Key genes associated with this pathway in the context of osseointegration include ligands (*Wnt3a*, *Wnt5a*, *Wnt10b*), receptors (*FZD*, *LRP5/6*), intracellular mediators (*CTNNB1 β-catenin*, AXIN2), transcription factors (*RUNX2*, T cell factor/lymphoid enhancer factor (*TCF/LEF*)), target genes (*ALPL*, *OCN*), and inhibitors (Sclerostin, Dickkopf-1 (*DKK1*)) (Perkins et al., 2023; Liao et al., 2022). The upregulation of osteogenic markers like *RUNX2*, *ALPL*, and *OCN* observed with various surface modifications is often mediated, at least in part, through the *Wnt* pathway (Jagannathan et al., 2024).

The understanding approach of Nuclear Factor kappa B (*NF-kB*) as a pivotal transcription factor family has been increasing in scientific evidence in recent years. The initial surgical trauma and the recognition of the implant as a foreign body trigger an inflammatory cascade. Triggered by pro-inflammatory cytokines (like *TNF*-α and *IL-1β*) or *TNF* receptor family members (like RANK), *NF-kB* signaling directly influences the expression of genes encoding numerous pro-inflammatory mediators, including *TNFα*, *IL-1β*, and *IL-6*. It also regulates the expression of *RANKL* and *OPG*, thus linking inflammation directly to bone remodeling (Emam and Moussa, 2024; Kandaswamy et al., 2024). Hence, implant surface characteristics were found to significantly modulate *NF-kB* activity. Titanium ions, for example, can activate *NF-kB* in adjacent macrophages, while rougher SLA surfaces were reported to evoke a stronger *NF-kB* mediated pro-inflammatory response compared to chemically modified hydrophilic SLActive surfaces (Gnanajothi and Rajasekar, 2024). Specific surface modifications can be designed to inhibit *NF-kB*; for instance, chitosan-gold nanoparticles delivering the transcription factor *c-myb* were shown to inhibit osteoclastogenesis by reducing *NF-kB* nuclear translocation (Jongrungsomran et al., 2024; Mahmood et al., 2024). Conversely, anodic oxidation of Titanium was found to increase the expression of *RANK* and *RANKL* suggesting enhanced *NF-kB* signaling potential via the *RANKL* axis. Furthermore, the literature has considered melatonin to suppress the *TLR4*/*NF-kB* pathway, potentially mitigating peri-implant inflammation (Wu et al., 2021). The consistent association of elevated pro-inflammatory cytokines (*TNF-α*, *IL-1β*, *IL-6*) with peri-implantitis and failure underscores the detrimental potential of sustained *NF-kB* activation (Mukaddam et al., 2025).

**FIGURE 4:**
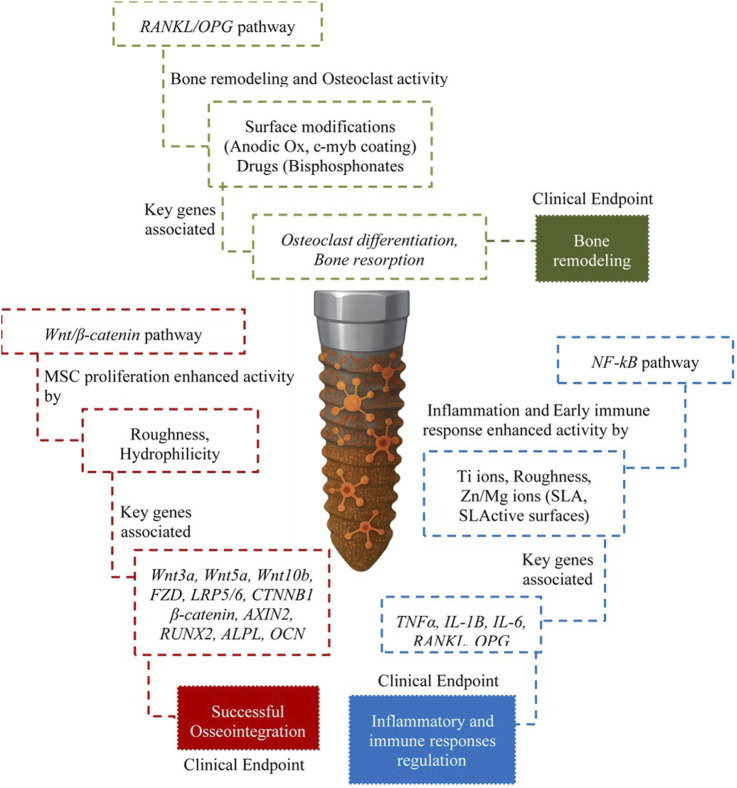
Surface features for *Wnt/β-catenin*, *NF-κB* and *RANKL/OPG* activity.

This inflammatory signaling directly impacts the *RANKL/RANK/OPG* system, the central axis controlling bone remodeling (Zhang C. et al., 2021; Saravi et al., 2021). The binding of *RANKL* to its receptor, *RANK*, initiates signaling (via *NF-kB* and MAPK) that promotes osteoclast survival, fusion, differentiation, and activation, leading to bone resorption (Freitag et al., 2023; Mukaddam et al., 2025; Zhang Z. et al., 2023). *OPG* has a special soluble function to decoy receptor that binds to *RANKL*, preventing its interaction with *RANK* and inhibiting osteoclastogenesis and bone resorption. Therefore, the balance between *RANKL* and *OPG* is critical for maintaining bone homeostasis at the implant interface. A high *RANKL*/*OPG* ratio favors net bone resorption, whereas a low ratio is related to bone formation or maintenance (Berk et al., 2022). Chronic inflammation consistently leads to an upregulation of *RANKL* expression coupled with a decrement in *OPG* production (Choukroun et al., 2024). Studies on failed implants confirm this, showing significantly decreased *OPG* expression and high *RANKL/OPG* ratios (Schluessel et al., 2022). Surface modifications can influence this axis, anodic oxidation of Titanium was shown to increase *RANK* and *RANKL* expression (Lv et al., 2022; Tao et al., 2025), whereas nanostructured Titanium surfaces can alter the secretion ratio of soluble *RANKL* (*sRANKL*) to *OPG* by macrophages (Fu et al., 2023; Mesa-Restrepo et al., 2024). Coatings delivering *c-myb* via chitosan-gold nanoparticles downregulated *RANKL* expression in osteoporotic models. Local delivery of bisphosphonates like alendronate from implant surfaces inhibits osteoclast differentiation, targeting the *RANKL* pathway (Mahmood et al., 2024; Sharifianjazi et al., 2022). Furthermore, genetic polymorphisms in the genes encoding *RANKL* (*TNFSF11*), *RANK* (*TNFRSF11A*), and *OPG* (*TNFRSF11B*) have been associated with increased susceptibility to peri-implantitis, highlighting the pathway’s clinical relevance (Xie et al., 2024; Huang et al., 2023). Figure 4 visually demonstrates the surface features for *Wnt/β-catenin*, *NF-kB* and *RANKL/OPG* activity.

A myriad of other signaling pathways intersects with and contribute to the complex molecular environment around dental implants. The *Nrf2* pathway’s activation leads to the expression of numerous antioxidant enzymes, combating oxidative stress (Jia et al., 2024; Schieffer et al., 2022). As oxidative stress is implicated in implant failure, *Nrf2* activation promotes bone formation and reduces inflammation (Choukroun et al., 2024; Addissouky et al., 2024). Likewise, the Salvador-Warts-Hippo together with the transcriptional co-activator Yes-associated protein (*YAP*), (*Hippo-YAP*) pathway is increasingly being recognized for its role in mechanotransduction and regulating stem cell fate, osteogenesis, angiogenesis, and osteoimmunology (Wang et al., 2024). When the pathway is inactive, it regulates *BMSCs* differentiation, osteoblast mineralization, osteoclast differentiation (potentially via *OPG* regulation), and angiogenesis (via *VEGF* and Notch interactions) (Zhou et al., 2020; Pan et al., 2024). It is of great relevance in its multifaceted role in osseointegration demonstrated by different research groups, as it noteworthy regulates Bone Marrow Stromal Cells (*BMSCs*) differentiation, osteoblast mineralization, osteoclast differentiation (potentially via *OPG* regulation), angiogenesis, and macrophage polarization (Zhou et al., 2020). A key result reported in this research area highlights the presence of Titanium ion toxicity, as it has been shown to impair osteogenesis via *YAP* dysregulation (Tang et al., 2021). Other studies have also made a significant distinction to the *TGF-β*/Smad pathway, where Zn-modified implant coatings have been demonstrated to enhance the osteogenic differentiation via this pathway (Zhang C. et al., 2021). Similarly, research articles have highlighted the influence of integrin binding, which triggers intracellular signaling cascades involving Focal Adhesion Kinase (*FAK*) on Titanium surfaces (Roy et al., 2024; Jung et al., 2020), where surface modifications like Mg ion incorporation can promote integrin expression (Zhang et al., 2025). The activation and effect of these pathways are highly context-dependent, varying with cell type and surface stimulus. Surface modifications are therefore a key tool to facilitate the timely transition from inflammation to bone-implant regeneration. Consequently, Table 5 exhibits the previously emphasized key signaling pathways modulated by dental implant surfaces (Han et al., 2025a; Choukroun et al., 2024; Zhou et al., 2020; Li et al., 2023; Tuikampee et al., 2024; Kandaswamy et al., 2024; Zhang et al., 2025; Zhang et al., 2024; Zhao et al., 2020; Alkhoury et al., 2020; Xu et al., 2023; Zhang J. et al., 2023; Abd Rashed et al., 2023; Li et al., 2024; Zieniewska et al., 2020; Abedi et al., 2023; Kumar et al., 2021; Wang J. et al., 2023; Liu et al., 2022; Ni, 2024; Bjelić and Finšgar, 2021; Wang et al., 2021c; Jin et al., 2024; Yang et al., 2022).

**TABLE 5:** Key signaling pathways modulated by dental implant surfaces.

Signaling Pathways	Primary Role in Osseointegration	Key Molecular Agents	Modulating Surface Features	Key Target Genes	References
*Wnt/β-catenin*	Osteogenesis, MSC/Osteoblast differentiation, Bone remodeling	*Wnt* ligands (*Wnt3A/5A/10b*, *Fz*, *LRP5/6*, β-catenin, *TCF*/*LEF*, Sclerostin, *DKK1*	Roughness, Hydrophilicity (via M2 macrophages), Wnt ligand delivery	*Runx2*↑, *ALPL*↑, *OCN*↑, *OPN*↑, *BSP*↑, Osteoblast differentiation↑, Bone formation↑	Zhang et al. (2024), Zhao et al. (2020)
*NF-kB*	Inflammation, Early immune response, Osteoclastogenesis link	*TNF-α*, *IL-1β*, *TLRs*, *IKKβ/α*, *IκBα*, *p50/RelA*, *p52/RelB*, *RANKL*, *OPG*	Ti ions, Roughness (SLA, SLActive), Chitosan-AuNP-c-myb coating↓, Anodic oxidation↑ (via *RANK/L*), Melatonin↓ (via TLR4), Zn/Mg ions↓ (via M2 promotion), GO coating↓	*TNFα*↑, *IL1B*↑, *IL6*↑, *RANKL*↑, *OPG*↓ (context-dependent), M1 macrophage activation↑, Osteoclastogenesis↑	Kandaswamy et al. (2024), Alkhoury et al. (2020), Xu et al. (2023)
*RANKL* *RANK* *OPG*	Bone remodeling, Osteoclast Differentiation and activation	*RANKL, RANK, OPG, TRAF6, NF-kB, MAPK*	Chronic inflammation↑ (*RANKL*↑, *OPG*↓), Surface mods (Anodic Ox↑, *c-myb* coating↓), Drugs (Bisphosphonates↓)	Osteoclast differentiation↑/↓, Bone resorption↑/↓ (dependent on *RANKL/OPG* ratio)	Tuikampee et al. (2024), Zhang et al. (2023c), Abd Rashed et al. (2023)
*Nrf2*	Antioxidant defense, Anti-inflammation, Bone formation support	*Nrf2, Keap1*	Vitamin C↑, Vitamin D↑, Potential for antioxidant coatings	Antioxidant enzyme genes↑ (*HO-1*, *NQO1*), Oxidative stress↓, Inflammation↓, Osteoblast apoptosis↓, Bone formation↑	Choukroun et al. (2024), Li et al. (2024), Zieniewska et al. (2020), Abedi et al. (2023)
*MAPK* *ERK*	Proliferation, Differentiation, Stress response, Apoptosis	*Ras, Raf, MEK, ERK, JNK, p38*	Ti particles↑, Oxidative stress↑, Growth factors↑ (downstream), GO coating↑ (*FAK/p38*), *PF-3845*↓ (*ERK*)	Cell proliferation/differentiation↑/↓, Osteoblast apoptosis↑ (if chronic/stress-induced), Inflammation↑ (p38), Osteoclastogenesis↑ (downstream of *RANKL*)	Li et al. (2023), Kumar et al. (2021), Wang et al. (2023b), Liu et al. (2022)
*Hippo-YAP*	Mechanotransduction, Organ size, Osteogenesis, Angiogenesis	*LATS1/2, MST1/2, YAP/TAZ, TEAD, LPA, VEGF*, Notch	Ti ions↓ (osteogenesis), Potential for topography-based modulation	Osteogenesis↑/↓, Angiogenesis↑, Osteoclastogenesis↓ (via *OPG*), Macrophage polarization *M1/M2* (conflicting data), Cell proliferation↑	Zhou et al. (2020), Ni (2024)
*TGF-β/Smad*	Osteogenesis, Cell differentiation, ECM production	*TGF-β* ligands, *TGF-β* Receptors, Smads (*Smad2/3/4*)	*TGF-β* delivery↑, Zn-modified coatings↑	*Runx2*↑, *ALPL*↑, *OCN*↑, Collagen synthesis↑, Osteoblast differentiation↑	Han et al. (2025a), Bjelić and Finšgar (2021), Wang et al. (2021c)
*Integrin/FAK*	Cell adhesion, Survival, Proliferation, Differentiation	Integrins (*αvβ3*), *FAK*, *PI3K*, *MAPK*	ECM protein adsorption (Fibronectin, Vitronectin), *RGD* peptides↑, Collagen coatings↑, Mg ions↑ (integrin expression)	Cell adhesion↑, Cell spreading↑, Activation of downstream pathways (*MAPK*, *PI3K/Akt*), Osteoblast differentiation↑	Zhang et al. (2025), Jin et al. (2024), Yang et al. (2022)

### 3.5 Risk of bias

**FIGURE 5:**
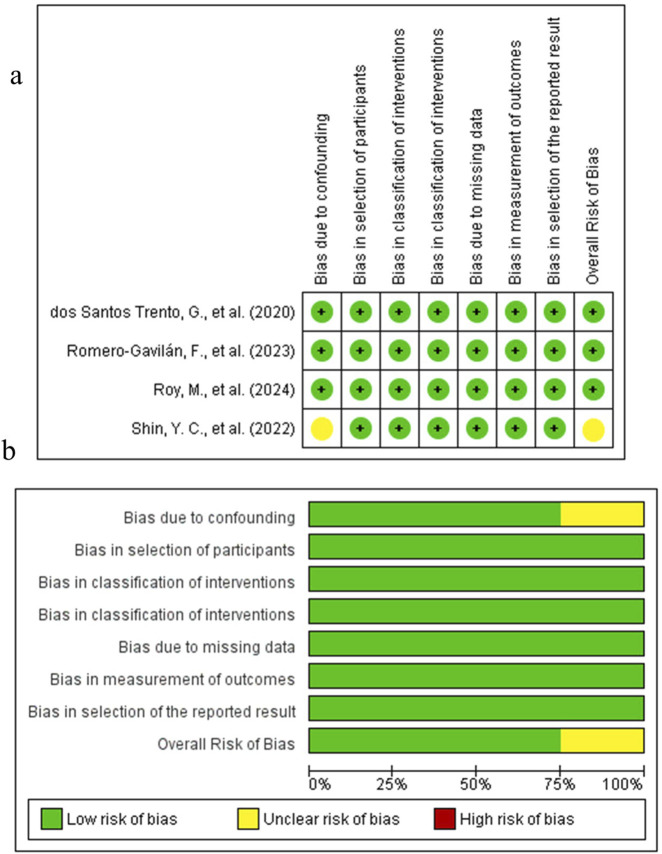
Risk of Bias Foundational Studies on Pro-Osseointegration Biomarkers. **(a)** Risk of Bias summary. **(b)** Risk of Bias graph.

**FIGURE 6:**
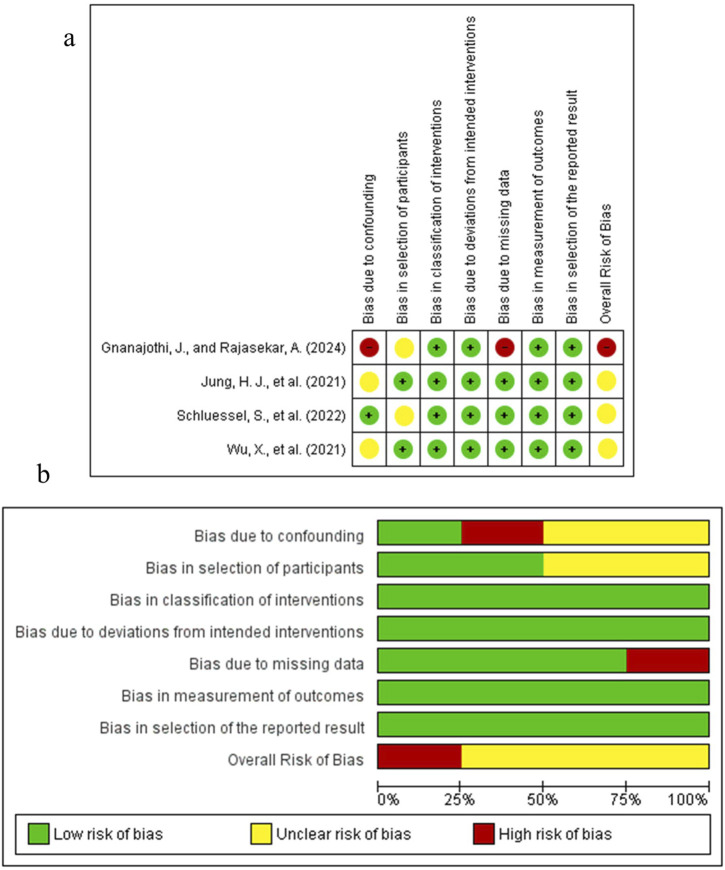
Key Studies on Inflammatory and Failure-Associated Pathways. **(a)** Risk of Bias summary. **(b)** Risk of Bias graph.

**FIGURE 7:**
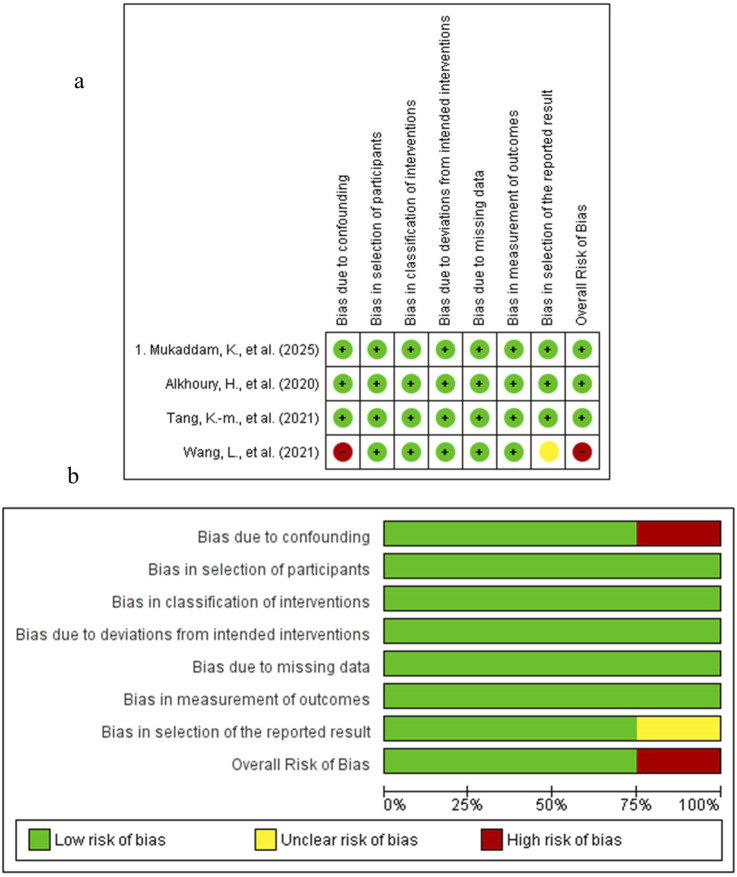
Studies on Key Signaling Pathways. **(a)** Risk of Bias summary. **(b)** Risk of Bias graph.

The results of the risk of bias assessment are depicted in Figures 5–7. The Figures were created using Risk-of-bias VISualization (ROBVis). For the single preclinical randomized trial by Shin et al. (2022), the overall judgment was “some concerns” of bias. This was due to concerns arising from the randomization process, as the method of sequence generation and allocation concealment was not described. The other domains for this study were judged to be at low risk of bias. For the non-randomized studies assessed with the ROBINS-I tool, several studies were judged to have a serious risk of bias. In this regard, the study by Gnanajothi and Rajasekar (2024), as the different implant types were not randomly allocated to patients, which could influence the inflammatory outcomes. Moreover, the study by Wang et al. (2021b), which compared a complex systemic disease model (Type II Diabetes Mellitus) with healthy controls without controlling for the numerous intrinsic differences between the groups. An uclear risk of bias was identified in several other studies. For instance, the study by Schluessel et al. (2022), as the cells were derived from a clinically heterogeneous patient population with failed implants. The animal studies by Jung et al. (2021) and Wu et al. (2021) were judged to be at moderate risk due to potential for unmeasured confounding between the intervention and control groups. The risk of bias for the other domains in these studies was generally low.

## 4 Future research directions

Dental implantology is continually evolving, striving for enhanced predictability and patient-specific solutions. The term implantogenomics is emerging within this pursuit, representing an interdisciplinary field that seeks to integrate genomics, molecular biology, and clinical dental implantology, with the particular aim of elucidating how an individual’s unique genetic and molecular responses influence the outcomes of dental implant procedures, particularly the osseointegration process (Albrektsson et al., 2023; Refai and Cochran, 2020). The current absence of this term as a formally established in high-impact, recent systematic reviews and research articles suggests it may be a nascent concept, perhaps more prevalent in broader scientific discussions or literature not meeting the specific inclusion criteria for this analysis, or a descriptor for an area of increasing research interest rather than a defined discipline (Emam and Moussa, 2024). The aim is to utilize this approach to design better implant healing strategies that perform predictably well across diverse patient populations, often by mitigating known risk factors. However, the precise trajectory of research, particularly in areas like osteo-immunomodulation and advanced biomaterial design, points towards a future where interventions may be more specific to individual molecular and immunological profiles, from a generalized approach to a more personalized one (Albrektsson et al., 2022). Functionalizing dental implant surfaces with bioactive coatings represents a prominent strategy to enhance osseointegration. The novelty in this field remains in the careful selection of specific Growth factors or their combinations, optimization of their concentrations, the choice of carrier materials for the coating, sophisticated methods of incorporation onto the implant surface, and the engineering of systems for controlled and sustained release of these biomolecules to achieve desired biological effects over time (van Oirschot et al., 2022). As detailed in section 3.2, various surface engineering strategies lead to the upregulation of osteogenic genes (*Runx2, OCN, ALPL, BSP, OPN, DMP1*); the mechanisms involve activation of specific signaling pathways (*SMAD* for BMPs, integrin signaling for ECM peptides) that converge on transcription factors regulating these genes. Regarding molecular and gene expression, studies consistently show that pathways related to immune response, bone metabolism, and oxidative stress are the most significantly affected when comparing peri-implant and periodontal tissues or healthy versus diseased states. In peri-implantitis, specific molecular signatures appear to differentiate it from periodontitis, potentially due to the influence of Titanium particles (Emam and Moussa, 2024). However, limitations in current research, including small sample sizes in many primary studies and insufficient publication of re-analyzable raw data, hinder comprehensive integrative meta-analyses of differentially expressed genes. Table 6 provides some key specific research approaches contributing to implant technology insights for future perspectives, focusing on molecular and gene expression details relevant to dental implantology and personalized therapy (Wang Z. et al., 2023; Morandini Rodrigues et al., 2022; Memenga-Nicksch et al., 2025; Wu et al., 2022).

**TABLE 6:** Summary of recent findings on personalized dental implantology.

Study Type	Focus of the Paper	Unique Contributions	Detailed Molecular Expression	Relevance to Personalized Dental Implant Therapy	References
Research	Nanoscale topographic modification of Titanium implants on *OSX* gene expression and osseointegration	Provides evidence of surface-associated gene regulation with a nanoscale osteoinductive pathway to direct adherent cell	Nanoscale surface adherent cells expressed higher levels of *OSX*, *ALPL*, Paired related homeobox 1 (*Prx1*), *DMP1*, *BSP*, and *OCN* compared to smooth surface implants over a 21-day healing period	Demonstrates how implant surface design can directly modulate host gene expression, offering a pathway for creating implants that actively promote personalized osseointegration at a molecular level	Morandini Rodrigues et al. (2022)
Review	Evaluation of Marginal one loss (MBL) comparing dental implants placed in augmented against pristine bone sites after ≥5 years	Provides long-term (5-year) MBL data, showing greater MBL in augmented sites, emphasizing the need for detailed description of patient and local factors to identify further risk factors	Discusses bone remodeling dynamics; MBL for pristine bone of 0.79 mm, compared to augmented bone of 1.90 mm. No specific gene expression detailed but points to biological processes influencing MBL.	Remarks the importance of considering patient-specific risk factors and local factors (beyond augmentation status) for personalized risk assessment to optimize long-term implant outcomes	Memenga-Nicksch et al. (2025)
Review	Summarize the impact of TiO2 nanotube arrays (NTAs) on Titanium implant surfaces on the stages of osseointegration, including protein adsorption, inflammatory response, cell adhesion, angiogenesis, and osteogenesis	Clarifies the impact of TiO2 NTAs on osseointegration in detail across four stages Proposes multi-dimensional regulation by TiO2 NTAs for efficient manipulation of osseointegration	TiO2 NTAs enhance macrophage adhesion and induce M2-polarization. Show ability to induce osteogenic differentiation and potential for blood vessel formation. Nanotubular layer can manipulate quantity, species, and conformation of adsorbed protein	Provides insights into how nanostructured surfaces can be rationally designed to modulate immune responses and promote osteogenesis, contributing to personalized implant surface engineering	Wu et al. (2022)
Review	Summarizes biomimetic approaches to improve osseointegration, including structural modifications and biomimetic coatings on dental implants	Discusses the importance of micro-nano-scale and porous structures, mimicking bone composition, and constructing functional coatings Highlights challenges in fabricating uniform biomimetic structures	Mentions that growth factors immobilized on implants pre-coated with calcium phosphate (*CaP*) are preferable for inducing bone formation and osseointegration. The immune microenvironment at the implant-bone interface should be considered in future designs	Contributes to the concept of personalized therapy by discussing how functional surfaces can be engineered to meet clinical needs and enhance osseointegration, considering the immune response	Wang et al. (2023a)

The integration of these latest scientific trends in molecular understanding into clinical practice involves several variables, mainly targeted to comprehensive patient evaluations, which include assessments of systemic disorders such as diabetes, osteoporosis, and cardiovascular diseases, current medication use, and lifestyle habits (particularly smoking) (Accioni et al., 2022). All these factors significantly influence implant survival rates and are often associated with distinct underlying molecular and cellular responses that can affect healing and long-term stability. Interestingly, even personality traits have been reported in initial research to correlate with implant favorable clinical rates in older patient populations, suggesting that psychological factors also have underlying molecular or behavioral correlates that impact treatment outcomes (Takefuji, 2025; Seki et al., 2022). The future direction, particularly highlighted in the literature analysis on immunomodulatory biomaterials, is towards personalized osseointegration therapies.

## 5 Discussion

Based on this systematic review, osseointegration is now understood to be an active and intricate biological process rather than a simple mechanical event. Therefore, the success of a dental implant fundamentally depends on a complex interplay of specific genetic and molecular signals initiated at the moment of placement. This view represents a critical departure from earlier, mechanics-focused models, marking a definitive shift toward a biological framework for evaluating implant outcomes. The rate of successful or failure performance in a dental implant is determined by a complex interplay between the host’s immune response, the genetic pathways of bone metabolism, and ultimately the physicochemical properties of the implant surface itself.

A critical finding reiterated across numerous studies is the temporal and sequential expression of key genes that drive bone formation. The process begins with hemostasis, where initial proteins (such as fibrinogen and fibronectin) form a scaffold that facilitates the recruitment of growth factors; including *PDGF*, *FGF*, and *VEGF* (Manor et al., 2021; Tavelli et al., 2024; Tavelli et al., 2023; La Monaca et al., 2023; Liu et al., 2021; Kuchinka et al., 2021; Kligman et al., 2021). This initial phase rapidly transitions to a crucial inflammatory response. The upregulation of a suite of osteogenic markers hallmarks the subsequent differentiation of osteoblasts. *RUNX2* emerges as a master regulator, orchestrating the expression of essential bone matrix proteins like Collagen 1, Bone Sialoprotein, and Osteocalcin (Hergem et al., 2022; dos Santos Trento et al., 2020; Le et al., 2021; Wang et al., 2020; Zhang W. et al., 2023). The reliable expression of these genes, particularly the early activity of Alkaline Phosphatase in promoting calcification, is a strong predictive indicator of a biological environment conducive to successful and robust osseointegration (da Cruz et al., 2022; Komatsu et al., 2025). Similarly, the interaction between *BMP* signaling, *DLX5*, and *RUNX2* represents a highly reported molecular mechanism in the signaling pathways that govern bone formation. These markers of bone regeneration play a crucial role in osteoblast proliferation, differentiation, bone osteogenesis, and remodeling; moreover, they remain vital genes during dental implant acceptance, from the very early to late stages. For an implant to achieve proper integration, the timely and sufficient expression of key pro-osteogenic factors is essential. The literature suggests that failures, by the same token, often have a clear molecular basis, typically originating from an inflammatory process that either fails to resolve or functions incorrectly. The development of peri-implantitis is strongly associated with the persistent high expression of pro-inflammatory cytokines, including *IL-1β, IL-6*, and *TNF-α* (Lumbikananda et al., 2024; Delucchi et al., 2023; La Monaca et al., 2025). These inflammatory mediators are not merely disease markers but also active participants in pathogenesis; directly contributing to bone loss by disrupting the critical balance of the *RANK/RANKL/OPG* signaling axis (Choukroun et al., 2024; Berk et al., 2022). An elevated *RANKL/OPG* ratio, driven by immune cells like T-lymphocytes, promotes osteoclastogenesis, leading to the progressive degradation of the bone-implant interface (Schluessel et al., 2022; Albrektsson et al., 2023). There is a strong basis for integrating personalized risk assessments into clinical implantology. This approach is justified by findings that specific genetic markers, particularly polymorphisms in the *IL-1* gene family, can make specific individuals susceptible to severe inflammation, a condition directly linked to a higher likelihood of peri-implantitis and implant loss (Lafuente-Ibáñez de Mendoza et al., 2022).

Regarding *in vitro* studies, they provide robust mechanistic evidence that the rGO coating actively promotes the differentiation of stem cells into bone-forming osteoblasts, establishing its osteoinductive potential at the most fundamental level (Shin et al., 2022). The *in vitro* setting allowed for a direct comparison between CGF-permeated implants and traditional uncoated implants. The findings clearly showed that CGF directly stimulated *hBMSCs* to increase the expression of *RUNX2*, *COL1a1*, and *OCN*. This insight is critical, as it confirms that the growth factors within CGF are biologically active and capable of inducing an osteogenic genetic program in human stem cells, providing a strong scientific rationale for its clinical use (Palermo et al., 2022). The most significant insight from Schluessel, S., et al. (2022) model was the identification of a distinct molecular signature of failure through RNA sequencing, the failure pattern was characterized by a dysregulated *RANKL/OPG* ratio and elevated TNF-α, which together create a microenvironment that drives the formation and activity of bone-resorbing osteoclasts. This finding provides a direct molecular explanation for the clinical observation of bone loss around failing implants. The study Shin, Y. C., et al. (2022) also provided the molecular promise seen *in vitro* translated directly to tangible structural benefits *in vivo*. Micro-CT and histomorphometric analyses confirmed that the rGO-coated implants resulted in significantly greater new bone formation compared to standard SLA surfaces.

In clinical human approaches one of the most significant insight was the identification of the chemokine *CCL5* as a biomarker that was explicitly overexpressed in areas of incomplete bone-to-implant contact. This finding is of high clinical importance as it moves beyond preclinical models to identify a molecular marker in human tissue that could potentially be used to assess the risk of poor osseointegration in patients (Lechner et al., 2024).

This systematic review and meta-analysis identified consistent molecular signatures associated with the success and failure of dental implants. The formal Risk of Bias (RoB) and GRADE assessments revealed important limitations within the current insights as there is an imperative need for methodological advancements in future research. The Risk of Bias assessment (summarized in Figures 5–7) indicated variability in the quality of the included primary studies. The single preclinical randomized trial was judged to have some concerns of bias, primarily due to insufficient reporting on the randomization and allocation concealment process. For the non-randomized studies, which constitute the majority of the evidence, the risk of bias ranged from low to serious. Several *in vitro* studies were well-controlled with a low risk of bias. However, several key studies were judged to have a moderate to severe risk of bias. This was most prominent in studies with a high potential for confounding; such as, the clinical study by Gnanajothi, J., and Rajasekar, A. (2024), where the non-randomized allocation of different commercial implants could significantly influence the inflammatory outcomes, and the preclinical study by Wang, L., et al. (2021). Furthermore, the narrative reviews included for their insights on signaling pathways were, as expected, rated as having critically low methodological quality by the AMSTAR-2 tool, as they lack a systematic search and formal bias appraisal. These study-level limitations directly inform the overall certainty of the evidence as evaluated by the GRADE framework (Table 6). The evidence for the key outcomes; the upregulation of pro-osteogenic markers, the association of pro-inflammatory cytokines with failure, and the role of the *RANKL/OPG* ratio, was consistently rated as Low certainty. This rating signifies that our confidence in the effect estimates is limited and that the actual effect may be substantially different from what is currently reported. This low certainty rating is the result of downgrading the evidence for three principal reasons; serious Risk of Bias as the evidence for each outcome is built upon the non-randomized studies detailed above, many of which carry a moderate to severe risk of bias from confounding and participant selection. Furthermore, there is significant clinical and statistical heterogeneity across the studies. For the pro-osteogenic markers, the meta-analysis of *RUNX2* expression revealed substantial statistical heterogeneity (I^2^ = 82%), which is a high value indicating that the observed effects vary significantly across studies, likely due to the vast diversity in the specific surface modifications (graphene oxide, CGF, ion-doping), cell lines, and experimental timelines employed. Finally, the finding suffered from serious imprecision as reflected by the wide 95% confidence interval for the pooled effect estimate (1.21–3.95). This range indicates considerable uncertainty about the true magnitude of the effect. Despite the current meta-analysis identifying a statistically significant and positive effect of modified surfaces on the master osteogenic pathway regulator, *RUNX2*, the very low certainty of the evidence means very little confidence in this effect estimate and the current effect is likely to be substantially different. This limitation likely extends to the qualitative findings for other pro-osteogenic and inflammatory pathways, as much of that evidence is derived from studies with similar preclinical designs. Therefore, the identified molecular signatures for success (*RUNX2* upregulation) and failure (pro-inflammatory cytokines and *RANKL/OPG* imbalance) should be viewed as strong, mechanistically plausible hypotheses that now require validation through higher-quality, standardized preclinical research and, ultimately, well-designed clinical trials.

The implant is far from being a passive witness in this biological process. As this review comprehensively details, the surface characteristics of a dental implant are a primary determinant of the host’s molecular response. Surface modification techniques, whether subtractive methods that alter topography or additive methods that apply bioactive coatings, are designed to actively steer cellular behavior toward osseointegration (Liu et al., 2020; Al-Zubaidi et al., 2020; Dong et al., 2020). Nanoscale surface features, for example, have been shown to directly enhance the expression of osteogenic genes, such as Osterix (Morandini Rodrigues et al., 2022). Modifications that increase hydrophilicity can promote more favorable initial protein adsorption and cellular attachment (Sadrkhah et al., 2023). These modifications exert their influence by modulating key intracellular signaling pathways. For instance, certain surfaces can promote an M2 macrophage phenotype that activates the *Wnt/β-catenin* pathway (a master regulator of bone formation) (Emam and Moussa, 2024; Tuikampee et al., 2024). In contrast, other surface properties or the release of metallic ions can trigger the *NF-kB* pathway, which, if chronically activated, perpetuates inflammation and bone resorption (Gnanajothi and Rajasekar, 2024; Mukaddam et al., 2025; Kandaswamy et al., 2024). This evidence demonstrates that implant design can be a powerful tool to program a desired biological outcome at the genetic level.

The formal Risk of Bias and GRADE assessments conducted in this review revealed that the overall certainty of the evidence for the key molecular signatures is Low. This rating is a result of downgrading as a result of serious risk of bias in the included non-randomized studies, significant clinical and statistical heterogeneity across different experimental models and surface types, and the preclinical evidence to human clinical outcomes. The focused analysis of *RUNX2* expression, revealed substantial statistical heterogeneity (I^2^ = 82%), likely due to the vast diversity in surface modifications and experimental protocols employed.

Ultimately, the literature synthesizes toward a future of personalized dental implantology, or implantogenomics, where treatment strategies are designed for the individual’s unique biological profile (Albrektsson et al., 2023; Refai and Cochran, 2020). By identifying and monitoring specific biomarkers in easily accessible oral fluids like GCF and PICF, clinicians could potentially gain real-time insights into the healing process, which may allow for early intervention before irreversible bone loss occurs (Gul et al., 2020; Zhou and Liu, 2023; Lumbikananda et al., 2024; Delucchi et al., 2023). However, this review confirms that the included studies do not provide the necessary performance data, such as sensitivity, specificity, or validated thresholds, to support their use as standalone diagnostic tools at present (Albrektsson et al., 2022; Accioni et al., 2022). Whereas the field has made significant strides, the low to moderate certainty of the current evidence underscores the need for more high-quality, long-term human clinical trials to validate these biomarkers and fully translate the potential of personalized, gene-centered implant therapy into standard clinical practice.

## 6 Conclusion

The field of dental implantology has undergone a fundamental evolution. As the evidence synthesized in this review demonstrates, there is a consensus that the biological and molecular processes driving osseointegration are the most critical factors for ensuring long-term clinical stability. The host’s response to an implant is not a passive mechanical process but an active, complex biological cascade governed by specific and predictable gene expression patterns. This review consolidates the evidence that successful osseointegration depends on a precisely timed sequence of molecular events beginning with an initial inflammatory response and transitioning into a robust osteogenic phase. A successful healing course is reliably indicated by the upregulated expression of principal transcription factors and the consequent synthesis of essential bone matrix proteins (including collagen, Osteocalcin, and Bone Sialoprotein). Conversely, implant failure and the loss of survival are often rooted in a dysregulated molecular environment characterized by a persistent pro-inflammatory state. The elevated expression of cytokines such as *IL-1β, IL-6, and TNF-α*, coupled with an imbalanced *RANKL/OPG* ratio, creates a microenvironment that favors osteoclast activity and progressive bone loss, leading to peri-implantitis. The literature strongly supports the concept that the surface of an implant has a powerful biological effect. Rather than being passive, it functions as a bioactive interface that appears to alter key signaling cascades, which govern cellular behavior and gene expression. Identifying specific gene expression patterns and biomarkers suggests their potential for monitoring osseointegration and for the early detection of pathological conditions. However, the current body of evidence, as highlighted by our GRADE assessment, is of low certainty and lacks the prospective validation and diagnostic performance data (sensitivity, specificity) required for clinical application. Integrating knowledge of a patient’s genetic predispositions with advanced immunomodulatory implant surface technologies may be possible to enable a move beyond a one-size-fits-all dental implant approach, but substantial research is required to translate these associative findings into validated clinical tools.

## Data Availability

The original contributions presented in the study are included in the article/Supplementary Material, further inquiries can be directed to the corresponding authors.

## Supplementary material

SUPPLEMENTARY TABLE S1 List of Studies Excluded at the Full-Text Screening Stage and Reasons for Exclusion.
